# Resource and Climate Implications of China’s Passenger Vehicle Fleet Transition to 2050

**DOI:** 10.1021/acs.est.6c01011

**Published:** 2026-08-06

**Authors:** Fanran Meng, Yiling Xiong, Hannes Gauch, Wei Ow, Xin Sun, Yi Feng, Shaojun Zhang, Jonathan M. Cullen

**Affiliations:** † School of Chemical, Materials and Biological Engineering, 7315 University of Sheffield, Mappin Street, Sheffield S1 3JD, United Kingdom; ‡ State Key Laboratory of Regional Environment and Sustainability, School of Environment, 12442 Tsinghua University, Beijing 100084, P. R. China; § Department of Engineering, University of Cambridge, Cambridge CB2 1PZ, United Kingdom; ∥ China Automotive Technology and Research Center Company, Ltd., No. 68 East Xianfeng Road, Dongli District, Tianjin 300300, China; ⊥ Beijing Laboratory of Environmental Frontier Technologies, Beijing 100084, P.R. China

**Keywords:** Fleet electrification, Vehicle stock, Sobol analysis, Material demand, Greenhouse gas emission

## Abstract

China’s rapid expansion and electrification of its passenger vehicle fleet will strongly influence global resource demand and greenhouse gas emissions. Yet existing projections rarely capture the complex, coupled effects of vehicle- and battery-related variables alongside fleet growth and decarbonization pathways. Here, we develop a bottom-up fleet stock model with exogenously specified powertrain adoption pathways to quantify China’s passenger vehicle stock and its greenhouse gas, energy, and material demand through 2050. The model is parametrized with China-specific data, evaluated against recent fleet statistics, and complemented by Monte Carlo, Sobol, and reference-scenario sensitivity analyses. We project that China’s passenger vehicle stock increases from 218 million in 2019 to 360–520 million by 2050, while life cycle greenhouse gas emissions, energy use, and material demand generally peak around 2035–2040 before stabilizing or declining. Depending on future behavioral and technological pathways, final energy demand and life-cycle greenhouse gas emissions range from approximately 53% below to 122–153% above the reference scenario. Sobol analysis shows that rebound effects account for 50–73% of output variance, followed by passenger travel demand (23–29%), whereas the timing of internal combustion engine phase-out contributes less than 0.5%. These findings demonstrate that a sustainable transport transition in China requires coordinated action on demand management, vehicle efficiency, electricity decarbonization, and material stewardship.

## Graphical abstract

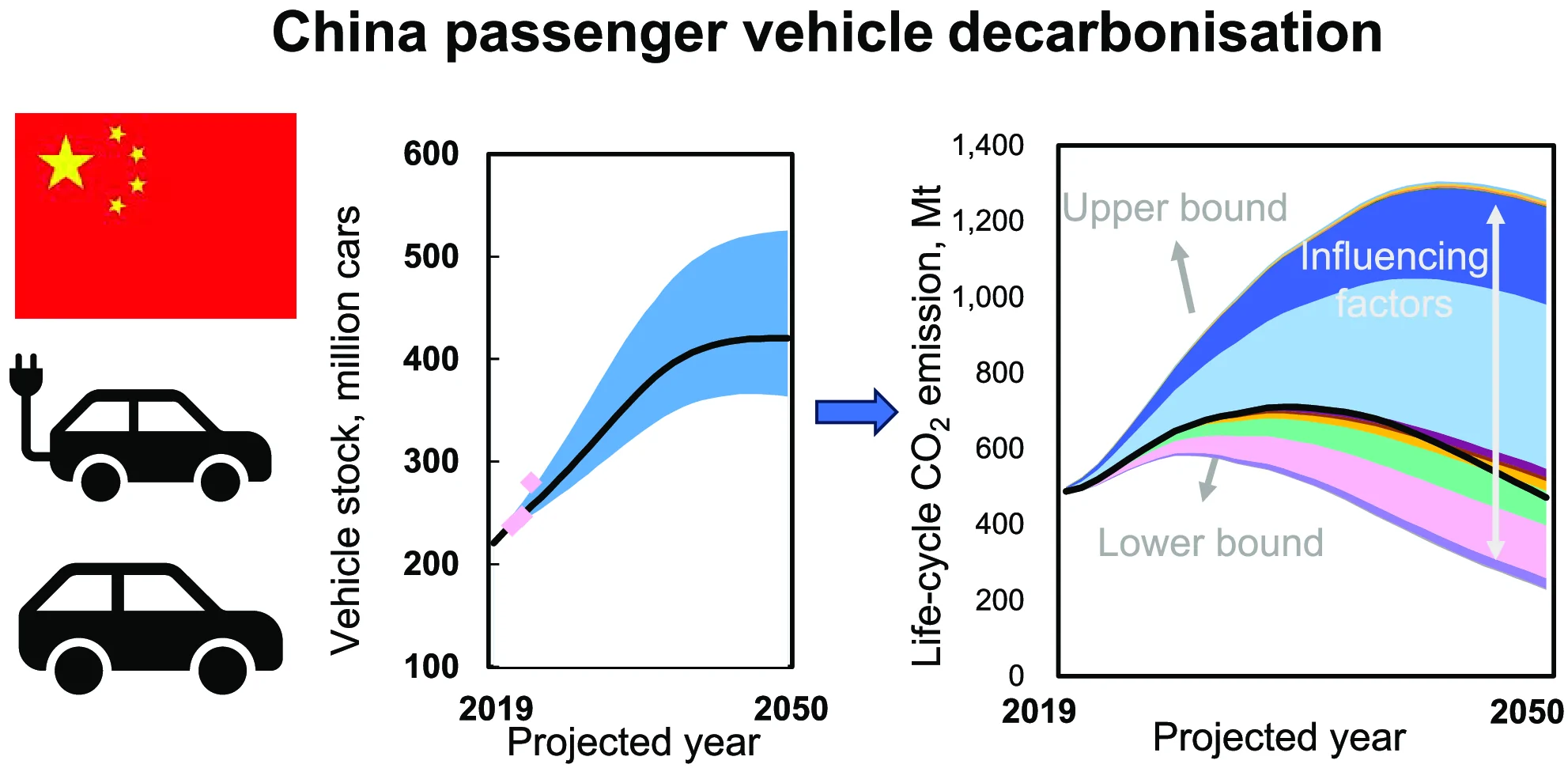

## 1 Introduction

The transport sector accounts for nearly one-quarter of global energy-related greenhouse gas (GHG) emissions and has been the fastest-growing end-use emitter over the past two decades.^1^ China, now the world’s largest automotive market, is undergoing a profound transformation as vehicle ownership surges alongside rapid electrification.^2^ While electric vehicles (EVs) are central to national and global decarbonization strategies, the pace and extent of achievable emission reductions, as well as the associated resource requirements, remain highly uncertain. The expansion of China’s vehicle fleet not only determines domestic energy and material flows but also exerts far-reaching implications for global supply chains of critical minerals and the trajectory of transport emissions.

Transport-related emissions arise from the complex interplay of multiple systemic factors. Previous studies have identified key drivers such as EV penetration, fuel consumption, carbon intensity of fuels, and vehicle miles traveled, all shaped by policy interventions, technological innovation, and consumer behavior.^3–5^ For instance, mandating 100% zero-emission vehicle sales has been identified as one of the most effective measures to reduce mobility-related emissions,^6^ while variations in driving behavior can significantly alter fuel consumption and GHG emissions from passenger cars.^7^ Collectively, strategies combining reduced travel demand, improved efficiency, and accelerated fleet electrification could lower transport emissions by 37–91% by 2050.^8^ However, most existing assessments primarily capture fuel-cycle impacts and neglect vehicle-cycle contributions or the dynamic and uncertain nature of evolving vehicle and battery technologies.

Critical parameters, including recycling rates, vehicle lifetimes, material composition, and battery energy density, are closely tied to vehicle and battery design and strongly influence both life cycle emissions and material demand.^9–11^ Milovanoff et al. integrated material composition into fleet GHG projections and showed that lightweight design could reduce cumulative emissions by 5.6% between 2016 and 2050.^12^ Zhu et al. highlighted the influence of recycling rates and lifetimes on truck-sector emissions,^9^ while Huo et al. compared fleet decarbonization across countries but treated vehicle weight as a fixed input, limiting the dynamic representation of vehicle-cycle impacts.^13^ Craglia and Cullen developed a bottom-up model incorporating nine uncertainty parameters to project energy use and CO_2_ emissions in the UK fleet,^14^ capturing changing vehicle attributes and technological improvement rates, yet still omitting uncertainties associated with EV-specific and battery-related parameters. Together, these studies underscore the absence of comprehensive, long-term projections that fully capture the evolving roles of vehicle and battery characteristics, factors essential for anticipating future resource needs and supply risks under accelerated decarbonization pathways.^15^

Scenario-based approaches remain the dominant paradigm for exploring transport decarbonization pathways,^9–11^ yet most rely on subjectively defined input parameters that depict idealized rather than plausible futures. There is a pressing need to assess the risks of future emissions and material demands by explicitly accounting for uncertainties and interactions among key variables.

Here we develop an integrated bottom-up fleet stock model that incorporates 28 interacting variables to project China’s light-duty passenger vehicle stock and its associated GHG emissions, energy use, and material demand through 2050. The model explicitly represents vehicle and battery attributes, travel demand, powertrain shares, and decarbonization measures, combined with Sobol sensitivity analysis to quantify their relative influence. Our results show that GHG emissions, energy use, and material demand from China’s vehicle fleet are likely to peak between 2035 and 2040 before stabilizing or declining. Managing overall fleet expansion emerges as a more decisive factor than the timing of conventional vehicle phase-out, as rebound effects from efficiency gains offset much of the emission benefit from electrification while intensifying material pressures. Beyond electricity decarbonization, vehicle attribute evolution, EV range, and battery energy density are identified as key levers shaping life cycle trajectories. These findings provide critical insights for mitigating future emissions and resource risks and guiding a sustainable transport transition.

## 2 Materials and Methods

### 2.1 China Fleet Electrification Impacts Projection Model

This study improves a fleet electrification impacts projection model based on previous work^14^ by incorporating additional EV and battery-related variables, material demand projections, and China-specific inputs. The model consists of seven interconnected modules: travel demand, stock, powertrain shares, fuel efficiency, vehicle weight, battery weight, and output (Figure 1). These modules are dynamically linked. The travel demand module determines required passenger kilometers, which are converted to required vehicle stock. The stock module then calculates fleet evolution using survival curves, which determine annual scrappage. Scrappage and required fleet size jointly determine new vehicle sales. The powertrain share module allocates new vehicle sales across technologies, which then influence energy consumption, material demand, and emissions through the fuel efficiency and vehicle/battery weight modules. The output module aggregates material requirements, final energy use, and life cycle greenhouse gas emissions.

**Figure 1:**
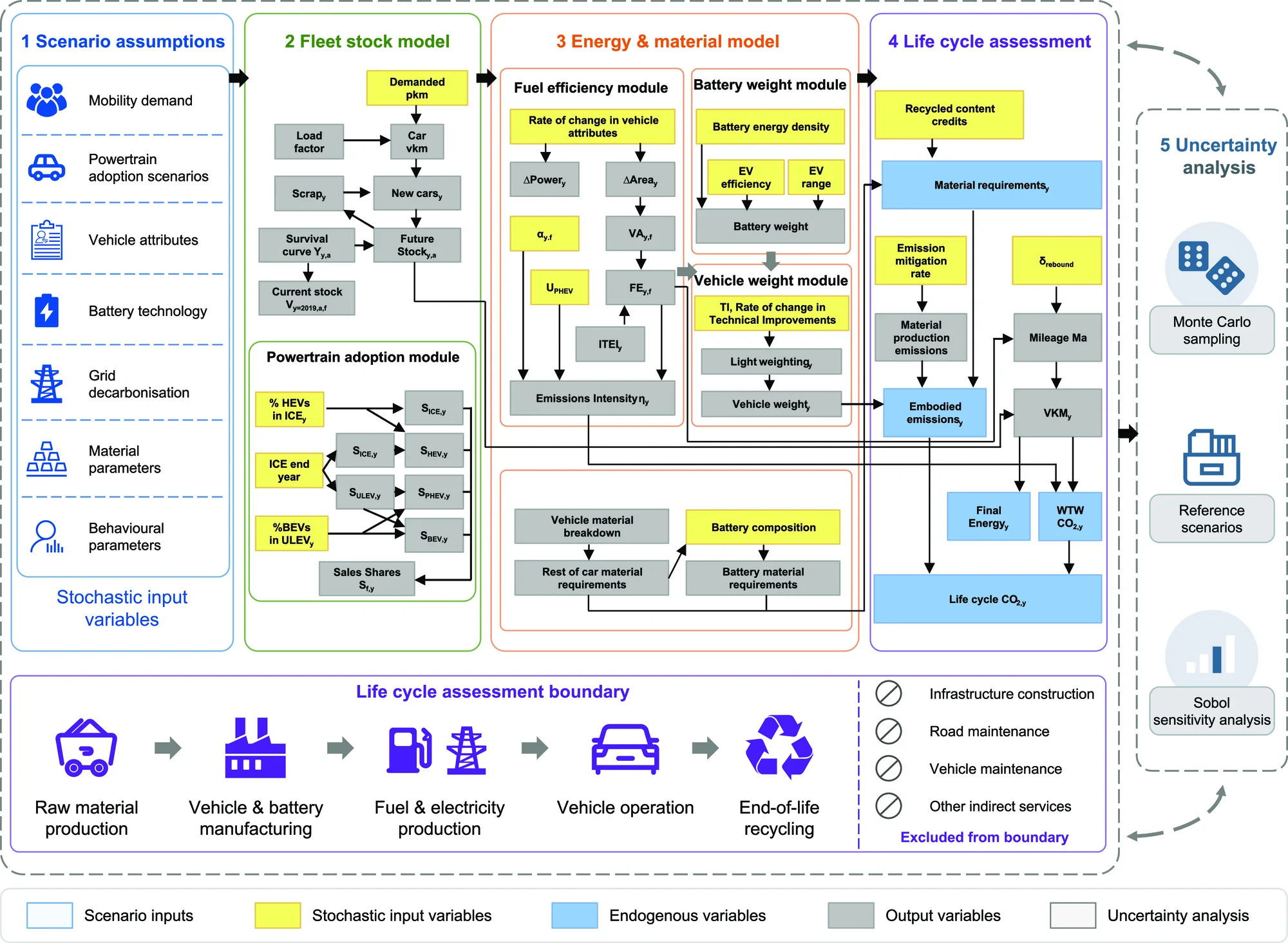
Overview of the integrated China passenger vehicle fleet projection model. The framework comprises five interconnected modules: (1) scenario assumptions, (2) fleet stock model, (3) energy and material model, (4) life cycle assessment module, and (5) uncertainty analysis. Scenario assumptions specify exogenous inputs describing future mobility demand, powertrain adoption, vehicle attributes, battery technology, electricity grid decarbonization, material parameters, and behavioral responses. The fleet stock model converts travel demand into vehicle stock using vehicle survival and scrappage functions, after which exogenously specified powertrain adoption pathways determine annual sales by powertrain. Vehicle attributes and battery characteristics determine vehicle energy consumption, battery size, and material requirements, which are combined to estimate final energy demand, well-to-wheel emissions, embodied emissions, material demand, and total life cycle greenhouse gas emissions. The life cycle assessment boundary includes raw material production, vehicle and battery manufacturing, fuel and electricity production, vehicle operation, and end-of-life recycling through recycled-content accounting, while infrastructure construction, road maintenance, vehicle maintenance, and other indirect services are excluded. Uncertainty is quantified using Monte Carlo sampling, reference-scenario analysis, and variance-based Sobol sensitivity analysis.

Each module contains stochastic input variables whose uncertainty ranges are localized to the Chinese context through consultation of national data sets, literature, and industrial databases. The full list of parameters and value ranges is provided in in the Supporting Information. Historical variables used for model calibration, including vehicle stock, vehicle sales, battery energy density, fleet composition, and vehicle mileage, are treated as deterministic inputs derived from observed data sources. Monte Carlo sampling is applied only to future projection parameters beyond the historical calibration period.

#### 2.1.1 Travel Demand Module

The travel demand module determines the total passenger-kilometers (pkm) required to meet future mobility needs. Owing to China’s rapidly evolving transport patterns, we replace the mode share input used in the UK version of the model^14^ with a stochastic variable for *demanded pkm*. According to the *China Statistical Yearbook*, total passenger transport in 2019 reached 3.5 trillion pkm across all modes, including 0.9 trillion pkm by highway.^16^ Unlike the stable travel patterns observed in mature markets, Chinese travel demand is expected to increase dynamically with economic growth, urbanization, and policy incentives. Based on data from the China Automotive Technology and Research Center Company (CATARC)^17^ and other studies,^18^ total passenger travel demand in 2050 is estimated to range between 4.5 and 10 trillion pkm. Population change is not modeled as a separate input variable. Instead, demographic effects are indirectly represented through the passenger travel demand scenarios, which reflect combined assumptions regarding population, income growth, urbanization, transport mode choice, and mobility behavior.

#### 2.1.2 Stock Module

The stock module projects annual vehicle stock and new vehicle demand required to satisfy travel needs. Vehicle survival is modeled using a modified Weibull distribution:^14^

1 $$\phi_{a}=\exp\left[-{\left(\frac{a+b}{T}\right)}^{b}\right]$$

where *a* is the vehicle age in years, *b* is the failure steepness of the curve and *T* is the characteristic service life. The values of *b* and *T* were updated for the Chinese model by considering China’s vehicle scrappage policies. Following China’s aggressive vehicle retirement policies, e.g., in 2009,[Bibr ref19] 2020,^20^ parameters were set to (*b* = 4, *T* = 18), consistent with an “austere scrappage” scenario analogous to Japan’s long-term survival rates.^21^ As shown in Figure S1, China exhibits the more aggressive scrappage rates in our model in comparison to countries with a higher proportion of older vehicles, such as the UK.

Vehicle stock in any year *y* is given by^14^

2 $$V_{y,a}=\frac{\phi_{a}}{\phi_{a-1}}\times V_{y-1,a-1}$$

where *V*_*y*,*a*_ and *V*_*y*–1,*a*–1_ are the numbers of vehicles aged *a* and *a* – 1 still on the road in the particular years *y* and *y* – 1, respectively. Vehicle-age data are initialized from CATARC, considering ages up to 10 years to reflect China’s rapidly evolving market and shorter vehicle lifetimes relative to Europe. This is because the Chinese vehicle market has undergone substantial changes with increasing vehicle ownership and passenger kilometers,^17^ and recent changes in scrappage policies. Hence, including older vehicle ages would not give an accurate reflection of long-term vehicle age distributions. Instead, an approximate model is developed that reflects the fleet when such factors are more stable.

Scrappage and new vehicle demand are calculated as^14^

3 $${Scrap}_{y}=\sum_{a}V_{y-1,a-1}\times \left(1-\frac{\phi_{a}}{\phi_{a-1}}\right)$$

4 $${\mathrm{New}\_\mathrm{Cars}}_{y}=\left(V_{y}-V_{y-1}\right)+{\mathrm{Scrap}}_{y}$$

where *V*_*y*_ is the demanded passenger kilometers in a given year *y*, which is calculated from the travel demand module. This model assumes that all cars being scrapped are replaced by the same number of new cars.

#### 2.1.3 Powertrain Shares Module

This module simulates the changing composition of the vehicle stock under fleet electrification. Baseline 2019 powertrain shares and internal combustion engine (ICE) phase-out schedules are aligned with CATARC data.^22^ A ten-year “grace period” is included to account for policy and market inertia. The powertrain shares module determines the composition of new vehicle sales.

#### 2.1.4 Fuel Efficiency Module

The fuel efficiency module estimates operational energy use and emissions by combining changes in vehicle attributes with evolving grid carbon intensity. Baseline 2019 fuel consumption values for each powertrain are sourced from the IEA, while attribute sensitivity coefficients follow the previous study.^23^ Electricity grid carbon intensity is projected using CATARC’s “Existing Policy” and “Enhanced Emission Reduction” scenarios, representing upper and lower bounds for electrification and decarbonization trajectories.^17^ Fuel consumption of new vehicles is projected from 2019 baseline values for each powertrain and adjusted over time according to incremental technical efficiency improvement (ITEI) and changes in vehicle attributes. For each powertrain *f* and model year *y*,

5 $$FE_{y,f}=FE_{2019,f}\cdot \mathrm{ITE}I_{y}^{\mathrm{FC}}+\Delta \mathrm{Powe}r_{y}\cdot \beta_{\mathrm{Power},f}+\Delta \mathrm{Are}a_{y}\cdot \beta_{\mathrm{Area},f}$$

where FE_2019,*f*_ is the baseline fuel consumption, ITEI_*y*_^FC^ is the cumulative fuel-efficiency improvement factor, ΔPower*_y_* and ΔArea_*y*_ are annual changes in vehicle power and frontal area, and β_Power,*f*_ and β_Area,*f*_ are sensitivity coefficients between vehicle attributes (power and frontal area).

EV efficiency improvement is represented as a bounded scenario parameter rather than an unbounded technological forecast. The 2050 EV efficiency range reflects plausible improvements from 2019 levels, while recognizing that efficiency gains may slow as vehicles approach practical design and performance limits.

For PHEVs, fuel consumption is modeled as a weighted average of combustion-mode and electric-mode operation:

6 $$FE_{y,\mathrm{PHEV}}=\left(1-U_{\mathrm{PHEV}}\right)FE_{y,\mathrm{PHEV},\mathrm{ICE}}+U_{\mathrm{PHEV}}FE_{y,\mathrm{PHEV},\mathrm{EV}}$$

where *U*_PHEV_ is the PHEV utilization factor. The PHEV utility factor is defined as the proportion of total PHEV driving distance traveled in electric mode. *A* value of 0 indicates exclusive combustion-engine operation, whereas a value of 1 indicates fully electric operation. In this study, the PHEV utility factor is varied to represent uncertainty in real-world charging behavior, charging access, trip length distribution, and electric-mode use.

Well-to-wheel emission intensity is then calculated as

7 $$g{\mathrm{CO}}_{2}\;km_{y,f}=FE_{y,f}\times EF_{y,f}$$

where EF_*y*,*f*_ is the fuel- or electricity-specific emission factor in year *y*.

#### 2.1.5 The Vehicle and Battery Weight Modules

These modules quantify material and energy requirements by estimating vehicle and battery weights, incorporating technological progress and design trends. Average 2019 vehicle weights by powertrain are derived from CATARC data. ITEI follows Craglia and Cullen^23^ and is assumed to remain consistent with international trends.

Battery weight is determined by three interacting stochastic variables: EV range, battery energy density, and EV efficiency. The baseline EV range (196 miles) and battery energy density (150 Wh kg^–1^) are based on 2019 market averages,^24^ with future ranges extended to 200–400 Wh kg^–1^ to capture technological uncertainty. Vehicle material composition is drawn from the GREET model, while battery material fractions are adapted from our recent study.^25^ EV efficiency is modeled using historic improvement rates of 1.7% yr^–1^ from 2000 to 2021 from the U.S. Department of Energy,^26^ with stochastic bounds of 1.0–1.5 times this rate to represent uncertainty in future advancements.

Battery weight is determined by energy density:

8 $$W_{\mathrm{bat},y}=\frac{E_{\mathrm{range},y}}{\rho_{\mathrm{bat},y}}$$

where *E*_range,*y*_ is the required energy and ρ_bat,*y*_ is the battery energy density.

Battery material composition is represented using bounded material fractions rather than explicit year-by-year battery chemistry market shares. The ranges for cobalt, nickel, and manganese contents capture uncertainty associated with shifts between nickel–cobalt–manganese-rich chemistries and lower-cobalt or LFP-like compositions. The model therefore represents the material implications of chemistry variation indirectly, rather than simulating an endogenous transition among specific battery chemistries.

Battery material demand is

9 $$\mathrm{Materia}l_{i,y}=W_{\mathrm{bat},y}\times c_{i}\times \mathrm{NewCar}s_{y,\mathrm{EV}}$$

where *c*_*i*_ is material composition for material *i*.

Total material demand is

10 $$\mathrm{Materia}l_{y}=\sum_{f}V_{y,f}\times m_{f}$$

#### 2.1.6 Output Module

The output module calculates material requirements, embodied emissions, and energy use, incorporating the rebound effect on vehicle mileage. Material-related emissions account for recycling, using upper and lower bounds derived from literature,^24,27–29^ and localized emission factors for aluminum, lithium, and graphite from Chinese industry data.^30–32^ Plastic, glass, and rubber recycling rates were not included in the model because of the challenges in recycling these materials from vehicles and the energy recovery from recycling these materials, over virgin production, is marginal.

Vehicle mileage is modeled as^14^

11 $$M_{y}=M_{y-1}\left[1+\delta_{\mathrm{rebound}}\times \frac{P_{y}-P_{y-1}}{P_{y}}\right]$$

where *M*_*y*_ and *M*_*y*–1_ are the vehicle mileages in years *y* and year *y* – 1, respectively; δ_rebound_ is the elasticity of vehicle mileage to fuel efficiency; and *P*_*y*_ and *P*_*y*–1_ are the average driving costs of years *y* and *y* – 1, respectively, and are determined by vehicle energy consumption and fuel or electricity prices. Driving cost includes fuel price, electricity price, and operating cost per kilometer. The rebound effect (δ_rebound_) is the elasticity of vehicle travel demand with respect to driving cost. δ_rebound_ represents the percentage increase in travel demand resulting from a 1% reduction in driving cost: a reduction in driving cost increases vehicle travel demand according to the rebound effect. For China, δ_rebound_ ranges from 0.26 to 0.82, derived from empirical studies of China’s transport sector,^35,36^ reflecting stronger behavioral responses compared with developed economies.^37^ Passenger travel demand specifies the overall mobility scenario, whereas rebound elasticity represents behavioral adjustment resulting from changes in generalized driving cost. These variables therefore describe different aspects of future mobility, although some interaction may exist in practice. Baseline mileage by vehicle age follows Ou et al.^34^ and declines from 10,700 to 6,165 miles yr^–1^ for new to 10-year-old cars.

Life cycle emissions are calculated as

12 $${\mathrm{CO}}_{2,y}={\mathrm{CO}}_{2,\mathrm{WTW},y}+{\mathrm{CO}}_{2,\mathrm{embodied},y}$$

### 2.2 Sobol Sensitivity Analysis

Variable sensitivity is evaluated using Sobol variance decomposition, which quantifies the contribution of each input variable *X*_*i*_ to the output variance *V*(*Y*) in terms of its Sobol index *S_Ti_*:^14^

13 $$S_{\mathrm{Ti}}=\frac{E_{X_{∼i}}\left(V_{X_{i}}\left(Y|X_{∼i}\right)\right)}{V\left(Y\right)}=1-\frac{V_{X_{∼i}}\left(E_{X_{i}}\left(Y|X_{∼i}\right)\right)}{V\left(Y\right)}$$

where *X*_∼*i*_ refers to all variables except *X*_*i*_. This effectively measures the impact of an input’s variance on that of the output. A total of 20,000 base samples were generated for each parameter set, resulting in 600,000 model evaluations. Monte Carlo simulations were performed by repeatedly sampling stochastic input variables within defined ranges (Tables S1 and S2), and Sobol indices were calculated once convergence in output variance was achieved. Convergence was assessed by monitoring stabilization of total-effect indices; indices varied by less than 2% when sample size increased by 20%, indicating convergence.

The uncertainty ranges presented in Figures 2–4 represent the combined effects of all uncertain inputs sampled simultaneously and should not be interpreted as measures of parameter importance. The magnitude of the uncertainty envelope depends partly on the assumed range of each input variable. Consequently, parameter importance is evaluated using variance-based Sobol sensitivity analysis rather than visual inspection of uncertainty bounds. Sobol indices quantify the contribution of each input variable and its interactions to total output variance across the full parameter space, providing a more robust assessment of influential drivers.

**Figure 2:**
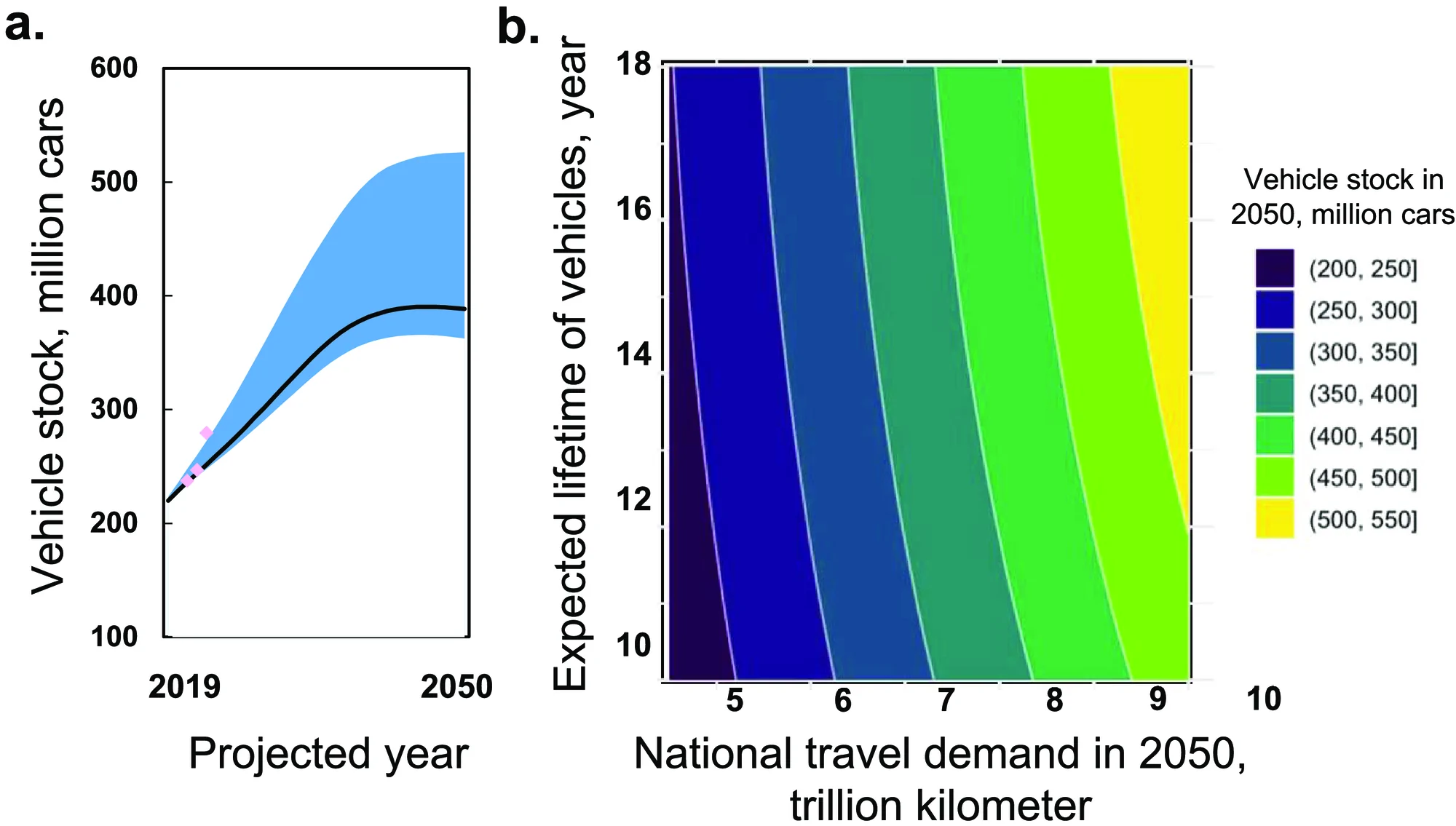
Vehicle stock projections and key stock drivers for China’s passenger vehicle fleet from 2019 to 2050. (a) Projected vehicle stock under the full uncertainty range of Monte Carlo simulations; the shaded region indicates the minimum–maximum range, the black solid line indicates the reference scenario based on central assumptions, and the pink points show observed vehicle stock values for 2021, 2022, and 2023 used for near-term validation. (b) Sensitivity of projected 2050 vehicle stock to two structural drivers: expected vehicle lifetime and national travel demand in 2050. The contour plot shows combinations of vehicle lifetime and travel demand resulting in similar vehicle stock levels. Full numerical values are provided in Tables SS1 and SS2.

### 2.3 Reference Scenario and Local Sensitivity Analysis

To enhance the interpretability of the uncertainty analysis, we define a reference scenario representing a central set of assumptions for key uncertain parameters. The reference scenario adopts midrange values for travel demand, rebound effect, electrification pathway, grid decarbonization, vehicle efficiency improvement, EV driving range, battery energy density, recycled content, and battery composition (see Table S3). This scenario is an assumed central projection used as a benchmark for model interpretation and local sensitivity analysis. It should not be interpreted as a prediction of the most likely future outcome.

Building on this reference case, we conduct a one-at-a-time sensitivity analysis, in which selected parameters are varied individually across their lower and upper bounds while all other parameters are held constant. This approach enables direct interpretation of the marginal influence of each parameter on cumulative life-cycle greenhouse gas emissions and cumulative final energy demand.

This local sensitivity analysis complements the Sobol analysis presented in Section 2.2. Whereas Sobol indices quantify the contribution of individual variables and their interactions to overall output variance across the full parameter space, one-at-a-time perturbations isolate the directional influence of each parameter relative to a consistent benchmark. The two approaches therefore provide complementary information: Sobol analysis identifies the dominant sources of uncertainty, while one-at-a-time sensitivity analysis reveals how individual assumptions increase or decrease cumulative energy demand and greenhouse gas emissions.

All uncertain parameters were represented using uniform probability distributions bounded by literature-derived minimum and maximum values (Table S2). Consistent with Craglia and Cullen,^33^ uniform distributions were selected because robust information regarding the future probability structure of many behavioral and technological variables is unavailable. This approach avoids imposing unsupported assumptions regarding central tendency or skewness and ensures equal sampling probability across the plausible parameter range. Consequently, the Monte Carlo analysis should be interpreted as exploratory uncertainty propagation rather than probabilistic forecasting. These uncertainty ranges are not applied retrospectively to historical data. Historical values such as vehicle stock, vehicle sales, battery energy density, fleet composition, and mileage are fixed model inputs obtained from statistical records, CATARC data sets, and published literature. All uncertain inputs were sampled independently. Potential correlations between technology, behavioral, and policy variables were not imposed because robust quantitative relationships are unavailable for long-term projections.

## 3 Results and Discussion

### 3.1 Future Fleet Characteristics

Figure 2 presents the projected evolution of China’s passenger vehicle stock and the main structural drivers embedded in the stock module. China’s passenger vehicle fleet is projected to expand substantially in the coming decades before stabilizing or slightly declining toward midcentury. Total vehicle stock increases from approximately 220 million vehicles in 2019 to around 360–520 million vehicles by 2050, depending on assumptions regarding travel demand and fleet turnover. The model links stock directly to required vehicle-kilometers and age-specific mileage, such that higher passenger travel demand requires a larger fleet to satisfy mobility demand. Longer vehicle lifetimes further increase the surviving stock, but their influence is weaker than that of national travel demand over the explored parameter range. The wide range of projected stock, therefore, reflects uncertainty in future passenger travel demand and vehicle usage intensity rather than population growth alone. To evaluate model reliability, we compared projections for vehicle stock and electric vehicle penetration against observed data for 2021–2023 obtained from CATARC. The observed stock values for 2021–2023 fall within the projected uncertainty envelope and closely follow the reference scenario, indicating that the stock module reproduces near-term fleet growth with reasonable accuracy. Figure 2a shows that the model reproduces recent trends in fleet growth and electrification. A detailed comparison is provided in Figure 2 and Table SS2, which shows observed and simulated values for vehicle stock.

Figure 2b clarifies this interaction. In contrast to the UK’s stable fleet projections, China’s fleet growth remains highly uncertain due to fluctuations in travel demand (Figure 2b). The Sobol sensitivity analysis reveals that vehicle stock is most sensitive to total demanded passenger-kilometers (pkm), while vehicle lifetime plays only a marginal role. For each additional 1 trillion km of travel demand, the 2050 fleet increases by about 50 million vehicles, a rise that cannot be offset even by extending vehicle lifetimes from 10 to 18 years. This result is important because stock size propagates through all downstream modules, affecting energy use, material demand, and life-cycle emissions.

From a technology perspective, the powertrain composition of the fleet depends primarily on the timing of ICE phase-out and the trajectory of EV market penetration. Assuming a linear transition, achieving an ICE phase-out by 2045 or 2035 would require EVs to capture 26% or 40% of market share by 2025, respectively. In practice, China’s EV market has already outpaced these trajectories, with new energy vehicle (NEV) sales exceeding 40% by 2025 and surpassing 50% in several months.^38^ This trend suggests that the transition to electrification is progressing faster than policy targets, although the implications for material and energy demand remain profound.

### 3.2 Material Requirements

Figure 3 shows projected annual material demand and the corresponding variance decomposition across the model inputs. Material demand trajectories are grouped into three categories according to their dominant application: (1) body-dominated materials such as steel, rubber, plastic, and glass; (2) battery-dominated materials such as lithium, graphite, nickel, cobalt, and manganese; and (3) mixed-use materials including aluminum and copper.

**Figure 3:**
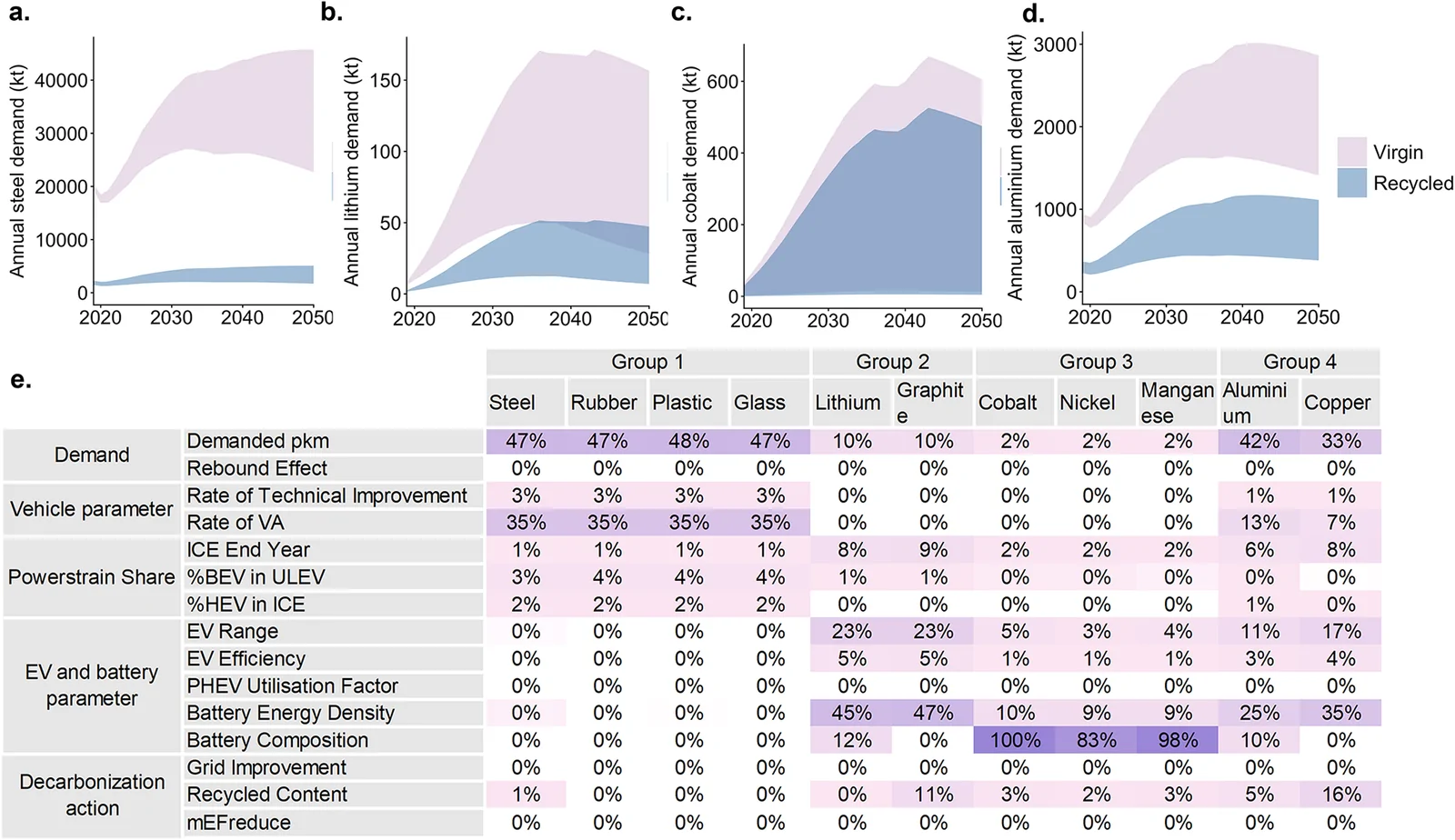
Material demand projections and sensitivity analysis for China’s passenger vehicle fleet from 2020 to 2050. Panels (a–d) show annual demand for steel, lithium, cobalt, and aluminum, respectively. In each panel, shaded bands indicate the full uncertainty range across Monte Carlo simulations. For metals with recycled supply shown explicitly, the lower shaded portion represents recycled material demand and the upper portion represents virgin material demand, such that the total stacked area gives overall annual demand. Panel (e) shows the total-effect Sobol indices for cumulative material demand, grouped by demand-side factors, vehicle attribute changes, powertrain share assumptions, EV and battery parameters, and decarbonization-related parameters. Larger percentages indicate a greater contribution of a parameter to output variance. The results show that steel demand is driven primarily by passenger travel demand, whereas lithium, cobalt, and aluminum are more strongly influenced by battery composition, battery energy density, EV range, and powertrain adoption assumptions. Numerical values are provided in Table SS3.

Body-dominated materials display relatively moderate changes over time. By 2050, annual demand is projected to range between 1.05 times and 2.33 times the 2019 level (Figures 3a and S3a–c). Growth before 2030 remains gradual (≤7% yr^–1^), driven by fleet expansion. After 2030, as total vehicle stock stabilizes, demand plateaus or slightly declines post-2040. These materials are primarily determined by the number of newly manufactured vehicles, since their usage per vehicle is relatively stable and unaffected by powertrain transitions. Steel remains the dominant material by mass throughout the study period because it scales primarily with total vehicle stock and average vehicle weight. As fleet size expands, annual steel demand rises steadily, although the magnitude of increase depends on assumptions regarding travel demand, vehicle attributes, and technical improvement. Recycled content modifies virgin steel demand, but the overall pattern remains governed by fleet expansion. The irregular fluctuations in the upper uncertainty bounds, particularly around 2040, arise from interactions among replacement cycles, exogenous powertrain adoption trajectories, and battery material requirements. Around this period, rapid ICE phase-out, EV market saturation, and replacement of earlier EV vintages occur simultaneously, producing nonsmooth changes in annual new vehicle demand and battery material requirements.

Battery-dominated materials, in contrast, exhibit substantial and multiphased growth. Critical battery materials such as lithium, cobalt, and nickel are controlled less by fleet size alone and more by electrification pathway and battery design assumptions. Their projected demand is shaped by the share of BEVs within ultralow-emission vehicles, EV range, battery energy density, and battery composition. Larger battery packs increase material requirements per vehicle, whereas improvements in battery energy density reduce material intensity for a given vehicle range. For example, demand for lithium and graphite, key active materials in Li-ion cells, rises sharply before 2030, with annual growth rates of 20 to 32%, driven by simultaneous increases in travel demand and EV adoption (Figures 3b and S3d). During 2030 to 2040, as ICEs are phased out and EV penetration matures, demand growth slows to 0 to 3% yr^–1^. After 2040, as the market saturates, demand begins to stabilize or decline. By 2050, lithium and graphite demand will reach 4 to 19 times their 2019 levels, even under conservative assumptions.

The irregular fluctuations in the upper uncertainty bounds, particularly around 2040, arise from interactions among replacement cycles, exogenous powertrain adoption trajectories, and battery material requirements. Around this period, rapid ICE phase-out, EV market saturation, and replacement of earlier EV vintages occur simultaneously, producing nonsmooth changes in annual new vehicle demand and battery material requirements.

Mixed-use materials, notably aluminum and copper, exhibit intermediate growth between body- and battery-dominated materials. Their increasing use in vehicle lightweighting and electrical components leads to cumulative demand growth of 2 to 5 times by 2050, with moderate uncertainty and sensitivity

Body-related materials show limited uncertainty due to stable design requirements and constrained variation in steel intensity (Figure 3e). Sobol indices indicate that demanded pkm contributes roughly 50% of total variance, followed by vehicle attribute rate (VA, 33%), which reflects the influence of vehicle size and power. In contrast, battery materials display significantly wider uncertainty ranges, reflecting technological volatility. For lithium and graphite, the battery energy density (34%) and EV range (27%) are the two most influential variables. This implies that battery design choices and user expectations for driving range will critically determine long-term material demand.

The uncertainty for cobalt, nickel, and manganese primarily reflects uncertainty in future EV battery chemistry pathways. Higher cobalt-, nickel-, and manganese-content assumptions approximate nickel–cobalt–manganese (NCM)-rich battery compositions, whereas lower values approximate a shift toward low-cobalt or lithium iron phosphate (LFP)-like chemistries. Consequently, demand for these metals varies substantially across scenarios: under NCM-rich assumptions, cumulative demand follows growth patterns similar to lithium, whereas a large-scale transition toward LFP-like chemistries can reduce cumulative cobalt and nickel demand by more than half. Because the model does not explicitly assign annual market shares to NCM and LFP batteries, these results should be interpreted as composition-bound sensitivity results rather than discrete chemistry-transition scenarios. For copper and aluminum, uncertainty is influenced by both battery requirements and broader vehicle design characteristics. The ICE end year contributes up to 5% of variance in China (compared with approximately 0.1% in the UK), reflecting the larger scale and faster pace of electrification in China’s vehicle market transition.

### 3.3 Energy Consumption and Greenhouse Gas Emissions

To complement the full uncertainty analysis, we define a reference scenario using central assumptions for travel demand, ICE phase-out timing, grid decarbonization, vehicle attribute growth, battery energy density, EV range, and recycled content. This scenario is not intended as a prediction; rather, it serves as a consistent benchmark reflecting plausible midrange development under current policy and technology trends. Comparing this benchmark with the uncertainty envelope helps identify which outcomes are robust and which remain highly contingent on future behavioral and technological change.

Figure 4 presents annual final energy use and life cycle GHG emissions under the full Monte Carlo ensemble, together with the reference scenario based on central input assumptions. The shaded envelopes represent the uncertainty range across simulations, while the reference case provides a policy-relevant benchmark for interpreting the effects of individual assumptions. Both energy use and life-cycle GHG emissions span wide uncertainty ranges. Depending on parameter combinations, energy use and emissions may decline by about 53% or increase by 122–153%. This wide solution space underscores the competing influences of mobility growth and decarbonization progress: improvements in energy efficiency and cleaner power generation could offset rising travel activity, but without these advances, total emissions may escalate.

**Figure 4:**
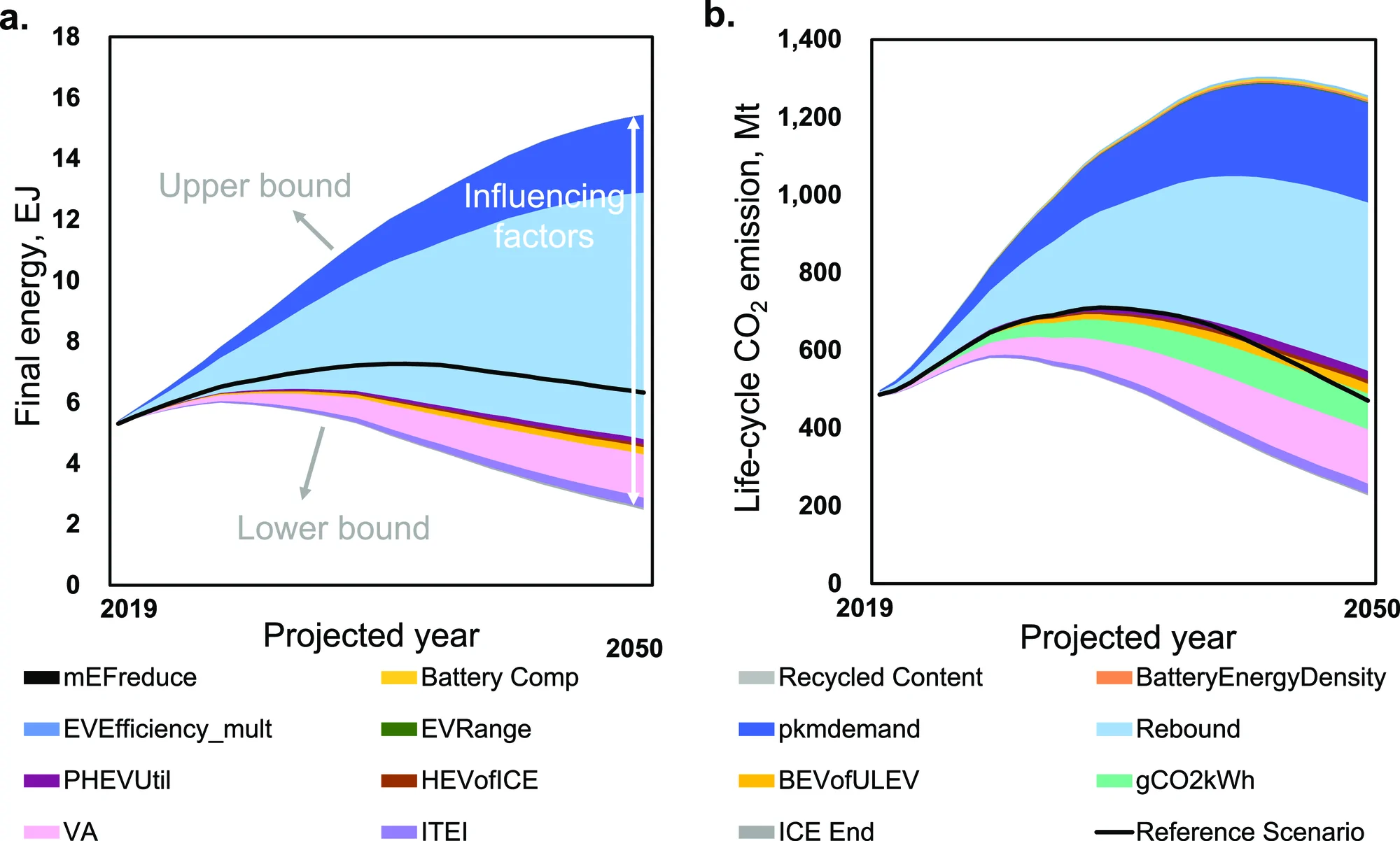
Projections of (a) final energy use and (b) life cycle greenhouse gas emissions for China’s passenger vehicle fleet (2020–2050).

Decomposing life cycle emissions reveals that WTW emissions currently account for about 80% of the total (Figure S4a), with embodied emissions, which arise from material production and manufacturing, comprising the remaining 20%. By 2050, embodied emissions rise to about 29%, driven by material-intensive EV production (Figure S4b).

The Sobol analysis identifies rebound effects as the most dominant driver, explaining 73% of the variance in final energy and 50% in life cycle GHG emissions. These importance rankings are derived from variance-based Sobol indices rather than from the width of the uncertainty ranges shown in Figure 4, thereby avoiding bias associated with differences in the assumed uncertainty ranges of individual inputs. The travel demand variable contributes another 23 to 29%, while vehicle attribute evolution accounts for 13 to 16%. The grid emission factor, representing the carbon intensity of electricity generation, contributes an additional about 11% to life cycle GHG uncertainty (see Figures S6 and S7).

Interestingly, the ICE end year exerts minimal influence on China’s total emissions (<0.5% of variance), in contrast to its substantial effect in mature markets. This suggests that China’s emission trajectory is governed less by phase-out pace than by demand growth, rebound behavior, and structural power-sector decarbonization. For embodied emissions, uncertainty is more evenly distributed among vehicle attributes, travel demand, battery composition, and energy density, reflecting the complexity of interactions between design and material supply.

A limitation of this study is that future EV efficiency is represented using bounded 2050 scenario values rather than an explicitly time-varying technology learning curve. Although this approach captures plausible long-term uncertainty, actual efficiency gains may slow over time as drivetrain, battery, and vehicle-design improvements approach technical limits. Future work could incorporate piecewise or saturation-based efficiency trajectories as more empirical data become available.

Despite wide uncertainty ranges, three findings remain robust across all simulations. First, travel demand and rebound effects dominate energy and emission outcomes. Second, battery energy density and EV range consistently control critical material demand. Third, the pace of ICE phase-out has limited influence on total emissions relative to demand growth. This result suggests that electrification alone is insufficient to guarantee deep decarbonization unless accompanied by demand restraint, efficiency improvement, and power-sector decarbonization.

The shaded regions represent minimum and maximum values across all Monte Carlo simulations. The black solid line shows the reference scenario based on central assumptions. Travel demand and rebound effect are identified as the dominant contributors to output variance based on Sobol sensitivity analysis, while grid carbon intensity contributes moderately. The timing of ICE phase-out shows limited influence compared to demand-driven factors. Full numerical values for the uncertainty ranges and sensitivity results are provided in Tables SS4 and SS5.

The reference scenario lies near the central tendency of the Monte Carlo ensemble for both cumulative life cycle emissions and final energy demand, indicating that it provides a representative benchmark for interpreting system behavior. One-at-a-time perturbation around this reference case (see Figure S5) shows that cumulative outcomes are most sensitive to travel demand and the rebound effect, while grid decarbonization exerts a stronger influence on emissions than on final energy demand. By contrast, variations in the timing of ICE phase-out have a comparatively smaller effect on cumulative results. These findings are consistent with the Sobol analysis but provide clearer directional insights into the role of individual parameters.

## 4 Discussion

Fleet electrification plays a central role in transport decarbonization and air quality improvement. Over 16 governments worldwide have proposed phase-out targets, aiming to achieve 100% zero-emission vehicle (ZEV) sales between 2030 and 2060.^39,40^ Previous studies have identified the phase-out time of ICE as a highly sensitive parameter for cumulative transport-sector emissions, underscoring its importance for accelerating decarbonization.^6,9^ However, these findings are largely based on relatively mature transport markets in developed countries. China’s vehicle market is simultaneously expanding and decarbonising, creating a complex interplay between rising mobility, fleet electrification, and resource constraints. Our integrated modeling and sensitivity analysis reveal that fleet scale, rebound effects, and technological attributes dominate the long-term dynamics of material demand, energy use, and emissions, overshadowing the effect of ICE phase-out timing. These conclusions are based on variance-based global sensitivity analysis and are therefore independent of the visual width of the uncertainty ranges reported in Figures 2–4. Future work could employ correlated sampling or scenario-dependent parameter relationships as improved evidence becomes available. Furthermore, the model is calibrated using China-specific data but represents the country as a single national system. Regional heterogeneity in travel demand, electricity generation, industrial structure, and vehicle ownership is therefore not captured.

The strong dependence of emissions and energy use on travel demand and rebound behavior highlights the urgency of curbing vehicle-kilometer growth. Rising efficiency and lower driving costs can induce greater travel, offsetting decarbonization gains. Policies promoting modal shift (public transport, active mobility) and demand management (road pricing, fuel taxes, or driving restrictions) are therefore critical. With rebound elasticity estimated at 0.26 to 0.82, China’s rebound effect is considerably higher than in developed economies, implying a stronger behavioral feedback that could erode the benefits of technological progress. Although passenger travel demand and rebound elasticity represent distinct processes in the model, some interaction may occur in reality, and future work could explicitly model their joint dependence.

While electricity decarbonization remains essential, as identified previously,^13^ its relative influence is constrained once a downward trajectory is established. The difference between moderate (54%) and deep (92%) grid decarbonization by 2050 explains only 8% of the variance in life cycle GHG emissions, equivalent to about 67 Mt CO_2_e in 2050. This reinforces that clean power must be paired with demand moderation and vehicle downsizing to achieve durable emission reductions.

As the fleet electrifies, the balance between vehicle downsizing and battery upscaling becomes pivotal. The VA rate emerges as a key driver of embodied emissions, suggesting that promoting smaller, lighter, and less powerful vehicles can substantially reduce material-related carbon footprints. For battery materials, energy density and range requirements are decisive: while higher energy density directly lowers material intensity, consumer preference for longer driving ranges can negate these gains. Expanding fast-charging infrastructure and urban charging networks could mitigate excessive range demand and help optimize material use.

For critical materials, particularly cobalt, supply chain risk is pronounced. Under high-demand trajectories, China’s cobalt requirement could reach 548 kt yr^–1^ by 2050, equivalent to 7.7% of current global reserves consumed annually by the automotive sector alone.^41^ However, rapid substitution toward LFP chemistries and low-cobalt NCM variants provides substantial mitigation potential. By 2025, the market share of LFP batteries in China is expected to exceed 60%, while the share of ternary batteries is declining sharply, from 40% in 2023 to about 20% in 2025. Battery chemistry transitions are represented through uncertain material composition ranges rather than explicit time-dependent NCM, LFP, or other chemistry shares. Future work could incorporate annual battery chemistry market-share trajectories to better represent the evolving Chinese battery market.

Several limitations merit attention. First, the Weibull scrappage model assumes fixed parameters, which may under- or overestimate retirements during early fleet transitions. Future work could treat scrappage coefficients as stochastic inputs or apply moving-average smoothing to reduce artifacts. Second, mileage profiles are assumed static over time, although emerging evidence indicates declining annual mileage as markets mature.^42^ Updating this relationship would enhance accuracy. Third, some data, particularly battery composition and recycling parameters, are drawn from UK or global sources, which could differ from China’s evolving industrial context. Finally, this model aggregates passenger vehicles and does not separately represent taxis or ride-hailing fleets, which typically have higher annual mileage and faster turnover. This simplification may affect absolute estimates of energy use and replacement demand, but sensitivity tests indicate that it does not materially change the ranking of the dominant drivers. Future expansion to commercial vehicles, buses, and freight would provide a more comprehensive picture of transport decarbonization.

## Supplementary Material





## Data Availability

All the data have been provided in the Supporting Information. Full codes are available from the corresponding author upon reasonable request.

## Notes

Numerical values underlying Figures 2–4 (Tables SS1–SS5) (XLSX)
China-specific model inputs (Table S1); ranges of stochastic input variables (Table S2); reference scenario inputs (Table S3); comparison of survival curves for the UK and China (Figure S1); comparison of vehicle stock trajectories between the two countries (Figure S2); annual material demand projections (Figure S3); projections of WTW and embodied CO_2_ emissions in China (Figure S4); one-at-a-time sensitivity analysis around the reference scenario (Figure S5); and Sobol sensitivity analysis results for cumulative greenhouse gas emissions and energy use (Figures S6 and S7) (PDF)
