# Supplementary material for: Resource and Climate Implications of China’s Passenger Vehicle Fleet Transition to 2050
Source: Environ Sci Technol. 2026 Aug 6;60(32):22335–44. doi: 10.1021/acs.est.6c01011 (PMC13492275; doi:10.1021/acs.est.6c01011)
Supplement: Supplementary file 1 [file es6c01011_si_002.pdf]

Supplementary information for

Resource and climate implications of China's passenger vehicle fleet transition to 2050

Fanran Meng[1]*, Yiling Xiong[1,2], Hannes Gauch[3], Wei Ow[3], Xin Sun[4], Yi Feng[4], Shaojun Zhang[2,5], Jonathan M. Cullen[3*]

[1] School of Chemical, Materials and Biological Engineering, University of Sheffield, Mappin St, Sheffield, S1 3JD UK

[2] State Key Laboratory of Regional Environment and Sustainability, and School of Environment, Tsinghua University, Beijing 100084, P. R. China

[3] Department of Engineering, University of Cambridge, Cambridge, CB2 1PZ, United Kingdom

[4] China Automotive Technology and Research Center Company, Ltd., No. 68 East Xianfeng Road, Dongli District, Tianjin 300300, China

[5] Beijing Laboratory of Environmental Frontier Technologies, Beijing 100084, China

*Correspondence to f.meng@sheffield.ac.uk; jmc99@cam.ac.uk

This Supporting Information contains:
Pages: 21
Figures: 8
Tables: 3

Nomenclature

Abbreviation / symbol	Full name / definition
BEV	Battery electric vehicle
CATARC	China Automotive Technology and Research Center
CO_2e	Carbon dioxide equivalent
EV	Electric vehicle
FC	Fuel consumption
FE	Fuel efficiency
GHG	Greenhouse gas
HEV	Hybrid electric vehicle
ICE	Internal combustion engine vehicle
IEA	International Energy Agency
ITEI	Incremental technical efficiency improvement
LFP	Lithium iron phosphate battery chemistry
NCM	Nickel–cobalt–manganese battery chemistry
OAT	One-at-a-time sensitivity analysis
PHEV	Plug-in hybrid electric vehicle
pkm	Passenger-kilometre
SI	Supporting Information
ULEV	Ultra-low emission vehicle
VA	Vehicle attributes
vkm / VKT	Vehicle-kilometres travelled
WTW	Well-to-wheel

S1 Summary of the localised items

Table S1 Summary of the localised items in this study

Item	Definition	Module	Source
Vehicle survivability curve	Weibull curve coefficients used to model age-specific vehicle survival and scrappage	Stock model	Huo et al.[1]
Current vehicle stock by age	Number of passenger vehicles in China by vehicle age in the base year	Stock model	CATARC [2]
Passenger travel demand in 2019	Passenger-kilometres travelled by passenger cars in China in the base year	Travel demand	CATARC [2]
2050 passenger travel demand range	Lower and upper bounds for passenger-kilometres travelled by passenger cars in 2050	Travel demand	CATARC[2]
Vehicle mileage by age	Average annual mileage of passenger vehicles by age in China	Travel demand	Ou et al. [3]
Initial ICE and EV shares	Shares of internal combustion engine vehicles and electric vehicles in the 2019 fleet	Powertrain shares	CATARC[2]
Initial HEV share in ICE fleet	Share of hybrid electric vehicles within the ICE-related fleet in 2019	Powertrain shares	CATARC[2]
Initial BEV share in ULEV fleet	Share of battery electric vehicles within ultra-low emission vehicles in 2019	Powertrain shares	CATARC[2]
ICE phase-out year	Assumed year by which new ICE vehicle sales reach zero	Powertrain shares	RFF report [4]
Grid carbon intensity projections	Projected electricity grid carbon intensity in China from 2019 to 2050	Fuel efficiency / emissions	CATARC[2]
Historical grid carbon intensity	Historical electricity grid carbon intensity in China	Fuel efficiency / emissions	IEA [5]
Fuel consumption by powertrain	Real-world fuel consumption of ICE, HEV, BEV and PHEV vehicles in 2019	Fuel efficiency	IEA[6]; CATARC[2]
Vehicle weight by powertrain	Average vehicle weight by powertrain type in 2019	Vehicle weight	CATARC[2]

Rebound Effect	Elasticity of travel demand with respect to driving cost	Travel demand / rebound	Zheng et al.[7]; Zhang et al[8].
Electricity and fuel prices	Electricity and petrol prices used to estimate driving cost	Rebound calculation	GlobalPetrolPrices.com
Battery energy density	Initial and future battery energy density assumptions	Battery module	CATARC[2]; technology roadmap/literature
EV range	Initial and future EV driving range assumptions	Battery module	CATARC; technology roadmap/literature
Battery composition	Material composition of EV batteries under alternative chemistry assumptions	Material demand	Literature[9]/industry data
Recycled content	Recycled content assumptions for vehicle and battery materials	Material demand	Literature[10]/industry data
Material emission factors	Emission factors for primary and recycled material production	Life-cycle emissions	LCA databases[11]

S2 Ranges of stochastic input variables for the China model

All uncertain parameters are sampled from uniform probability distributions bounded by literature-derived minimum and maximum values. Uniform distributions were selected because insufficient evidence exists to justify alternative distributional forms for long-term technological and behavioural variables.

Table S2 Ranges of stochastic input variables[a]

**Input**	**Definition**	**Distribution**	**Range**	**Basis for range**	**Source**
ICE End Year	Year when ICEs are banned	Uniform	2035 to 2045 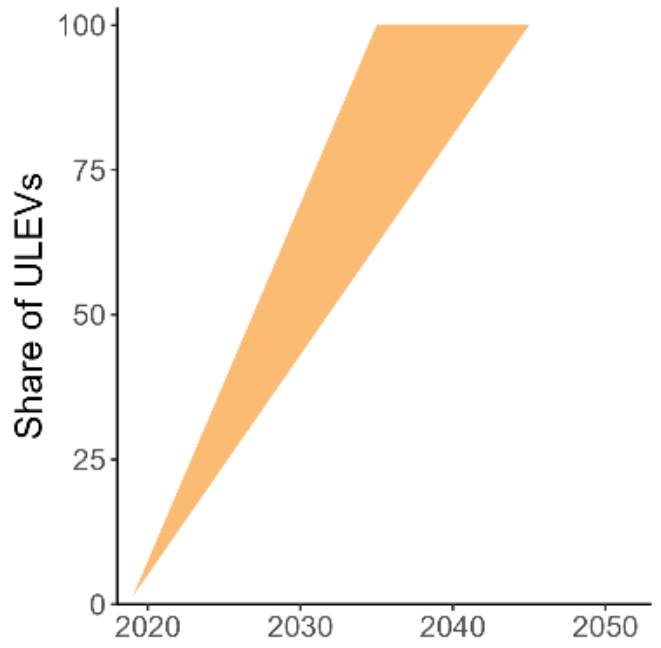	Conservative to ambitious ICE phase-out assumptions	RFF report
Rate of Technical Improvements	Multiplier applied to historical annual vehicle energy-efficiency improvement	Uniform	0.5 to 1.5 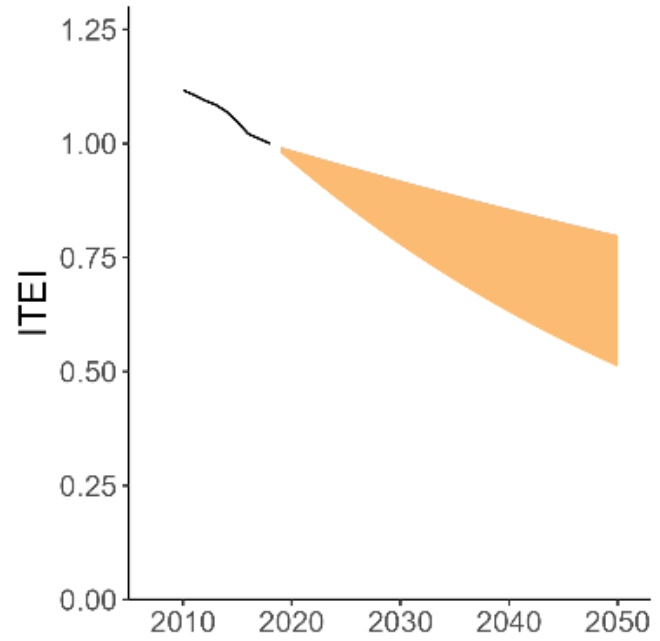	Slower-to-faster future efficiency improvement relative to historical trend	Historical data; technology literature
Rate of vehicle attributes change	Multiplier applied to historical change in vehicle power and frontal area	Uniform	-1 to 1.25	Possible lightweighting/down-sizing to continued growth in vehicle attributes	Historical data; vehicle attribute analysis

			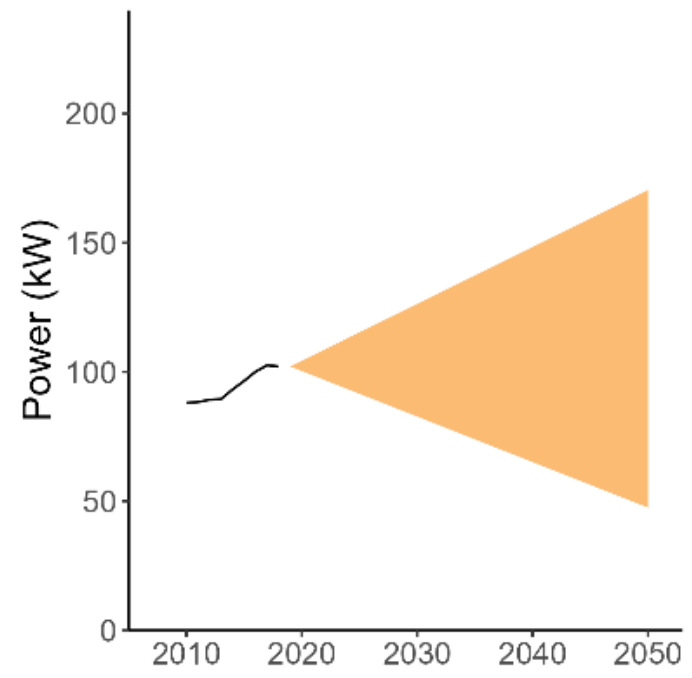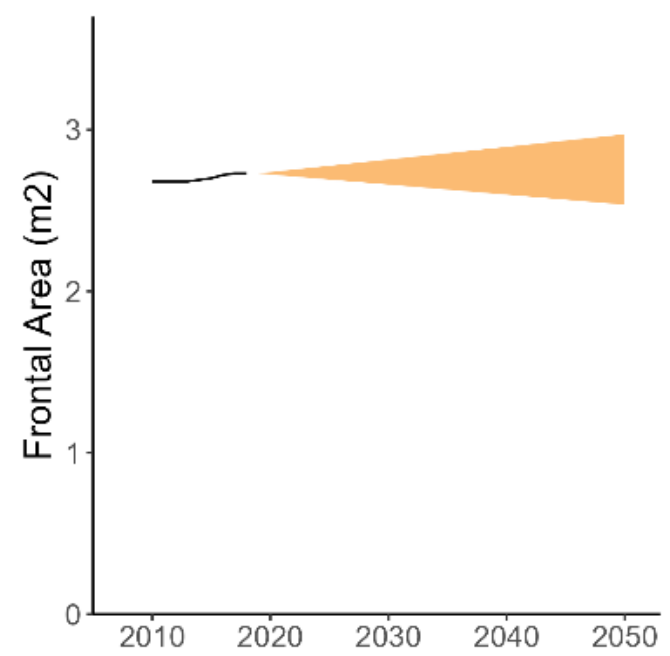		
Grid Improvement	Rate of improvement of electricity grid (gCO_2/kWh)	Uniform	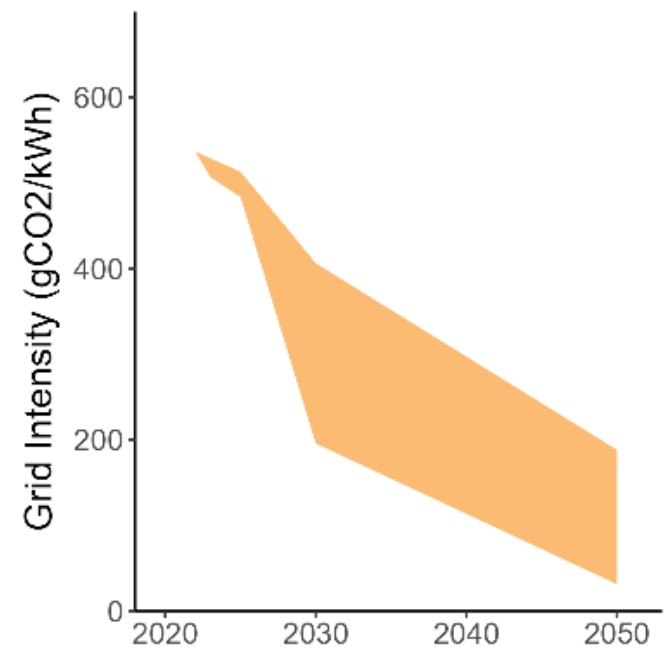	Range between conservative and enhanced grid decarbonisation pathways	CATARC

BEV share in ULEV	Share of BEVs within ultra-low emission vehicle sales in 2050	Uniform	0.2 to 0.8<br>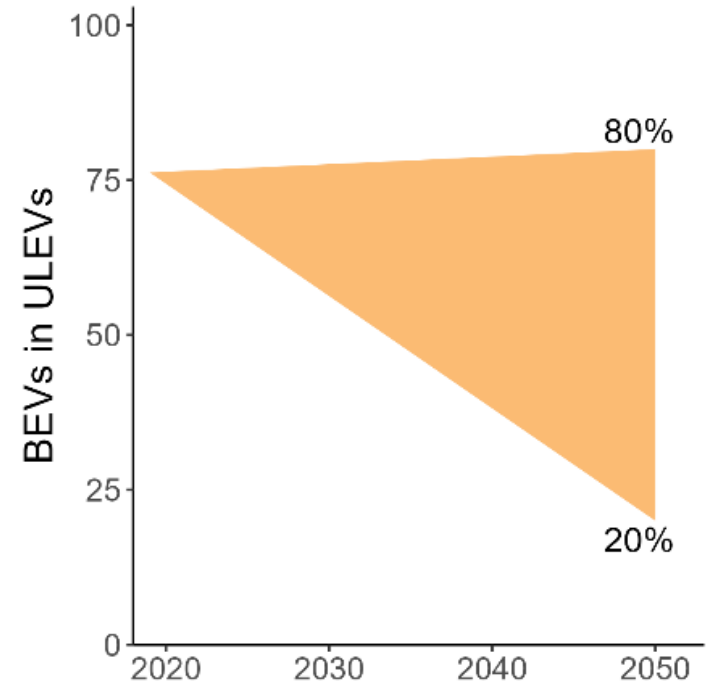	Range from PHEV-dominant to BEV-dominant electrification pathways	CATARC; scenario assumption
HEV share in ICE	Share of HEVs within remaining ICE-related sales in 2050	Uniform	0.2 to 0.8<br>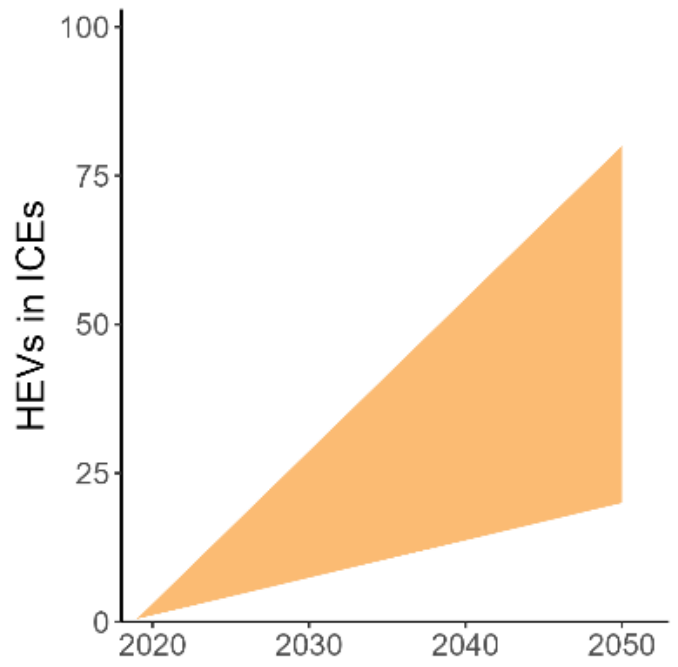	Range from conventional ICE-dominant to hybrid-dominant transition pathways	CATARC; scenario assumption
PHEV Utilization Factor	Proportion of PHEV driving distance travelled in electric mode	Uniform	0.2 to 0.5	Reflects uncertainty in charging behaviour, access to charging, trip length and electric-mode use	Literature on PHEV real-world use

Rebound effect	Elasticity of travel demand with respect to driving cost	Uniform	0.26 to 0.82	Lower and upper empirical estimates for China's transport rebound effect	Zheng et al.; Zhang et al.
Passenger travel demand	Passenger-kilometres travelled by passenger cars in 2050 (trillion kilometer)	Uniform	4.5 to 10	Lower and upper national mobility demand scenarios	CATARC
EV Range	Electric vehicle driving range in 2050 (miles)	Uniform	200 to 350 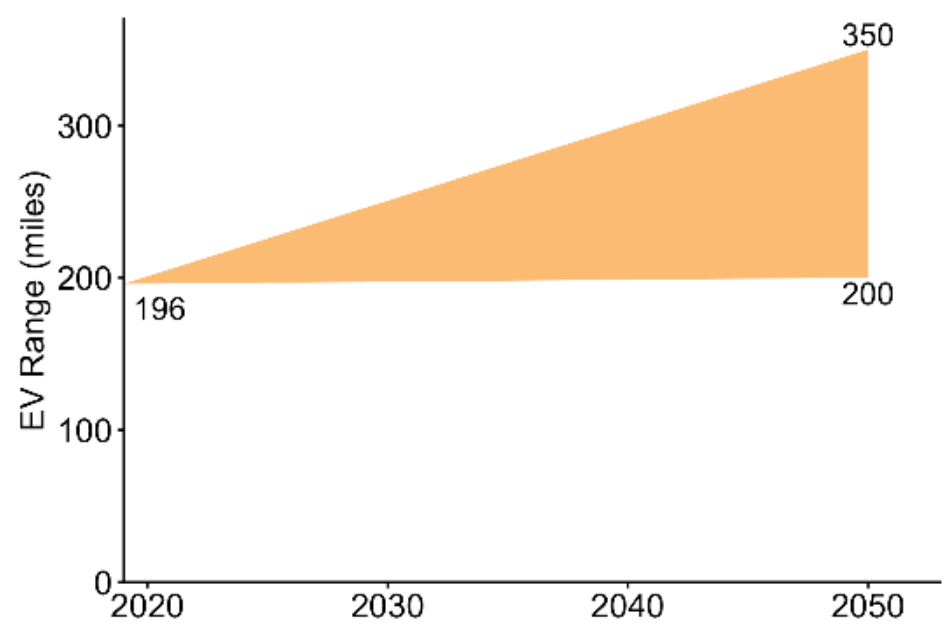	Conservative to ambitious range improvement assumptions	CATARC; technology roadmap/literature
EV Efficiency improvement	Relative improvement in EV efficiency in 2050 compared with 2019; treated as a bounded scenario parameter, not an unbounded	Uniform	1 to 1.5	No improvement to moderate long-term drivetrain efficiency improvement	Historical data; engineering assessment

	compound improvement forecast.		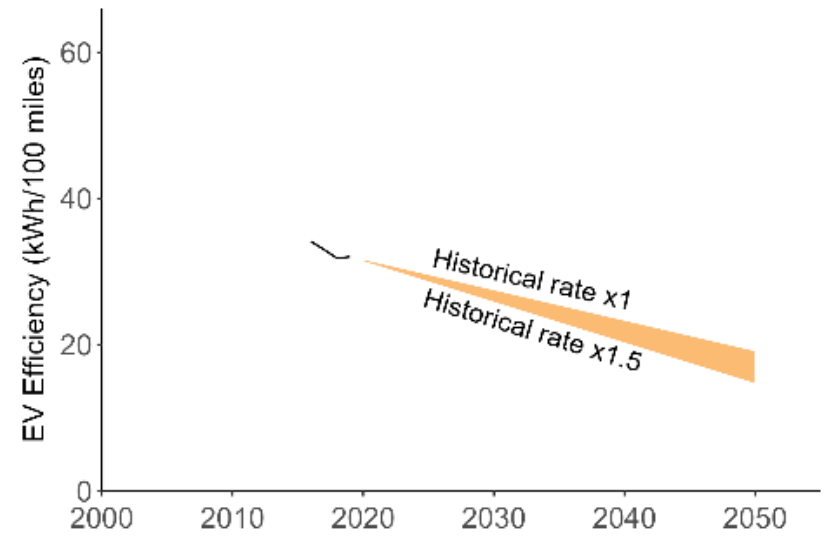		
Battery Energy Density	Battery energy density in 2050 (Wh/kg)	Uniform	200 to 400<br>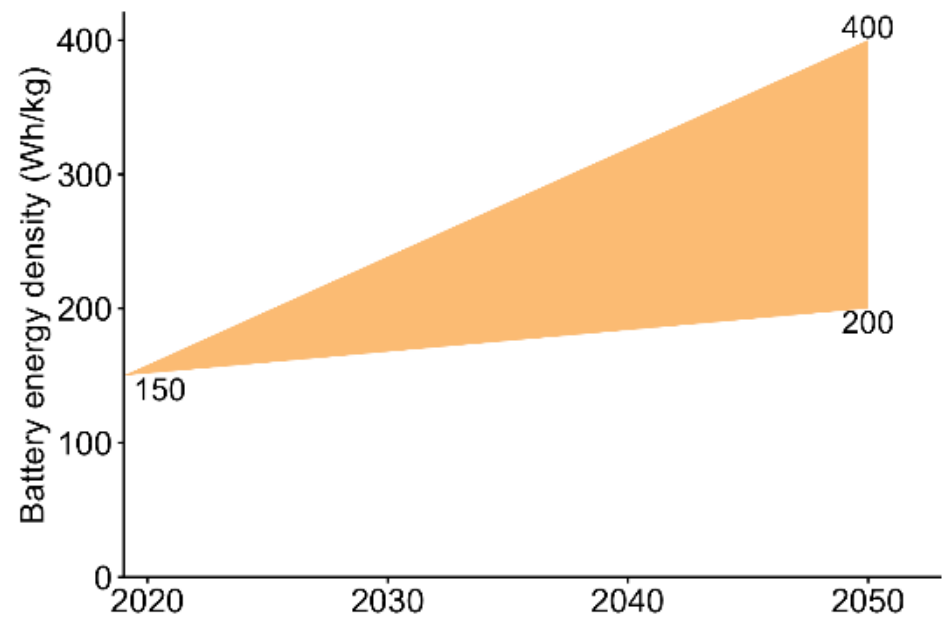	Conservative to ambitious battery technology development	Technology roadmap/literature
Steel Recycled Content	Recycled content of steel used in vehicle production	Uniform	0.07 to 0.1	Current and improved recycled-content assumptions	Literature/industry data
Lithium Recycled Content	Recycled content of lithium used in battery production	Uniform	0.199 to 0.232	Current and improved recycling assumptions	Literature/industry data
Cobalt Recycled Content	Recycled content of cobalt used in battery production	Uniform	0.263 to 0.440	Current and improved recycling assumptions	Literature/industry data

Aluminum Recycled Content	Recycled content of aluminium used in vehicle/battery production	Uniform	0.212 to 0.280	Current and improved recycling assumptions	Literature/industry data
Copper Recycled Content	Recycled content of copper used in vehicle/battery production	Uniform	0.213 to 0.317	Current and improved recycling assumptions	Literature/industry data
Nickel Recycled Content	Recycled content of nickel used in battery production	Uniform	0.222 to 0.377	Current and improved recycling assumptions	Literature/industry data
Manganese Recycled Content	Recycled content of manganese used in battery production	Uniform	0.259 to 0.441	Current and improved recycling assumptions	Literature/industry data
Graphite Recycled Content	Recycled content of graphite used in battery production	Uniform	0.215 to 0.382	Current and improved recycling assumptions	Literature/industry data
Steel Battery Composition	Steel mass fraction in battery pack	Uniform	0.1402 to 0.160	Range across battery pack composition assumptions	Literature/industry data
Lithium Battery Composition	Lithium mass fraction in battery pack	Uniform	0.0160 to 0.0198	Range across battery chemistry assumptions	Literature/industry data
Cobalt Battery Composition	Cobalt mass fraction in battery pack	Uniform	0 to 0.1056	Range from cobalt-free to cobalt-containing chemistries	Literature/industry data

Aluminum Battery Composition	Aluminium mass fraction in battery pack	Uniform	0.14 to 0.1888	Range across battery pack composition assumptions	Literature/industry data
Nickel Battery Composition	Nickel mass fraction in battery pack	Uniform	0 to 0.288	Range from nickel-free to nickel-rich chemistries	Literature/industry data
Manganese Battery Composition	Manganese mass fraction in battery pack	Uniform	0 to 0.304	Range across battery chemistry assumptions	Literature/industry data
Material Emission Factor Mitigation	Reduction in emission factors for lithium, cobalt, aluminium and synthetic graphite production by 2050	Uniform	0 to 80%	Conservative to ambitious decarbonisation of material production	LCA literature; decarbonisation scenarios

[a] The variable ranges not shown in the figures indicate that the uncertainty remains within this range throughout 2019–2050 in the projection model.

The uncertainty bounds were compiled from national statistics, CATARC projections, peer-reviewed studies, and technology roadmaps. Where multiple sources were available, the lower and upper bounds correspond to conservative and ambitious development pathways reported in the literature. Behavioural parameters (e.g. rebound effect and travel demand) were derived from published empirical studies, while technological parameters (e.g. battery energy density and EV efficiency) were based on industry outlooks and engineering assessments. The ranges, therefore, represent plausible futures rather than statistical confidence intervals.

Historical variables, including vehicle stock, vehicle sales, battery energy density, and fleet composition, are treated as deterministic inputs obtained from statistical records and industry datasets. Uncertainty is only applied to future projections beyond the calibration period.

S3 Reference scenario assumptions

Table S3 Reference scenario inputs

**Inputs**	**Values**
ICEEnd_Year	2040
ITEI	1
VA_mult	0.25
gridinc	0.5
BEVofULEV	0.7
HEVofICE	0.4
PHEVUtil	0.35
Rebound	-0.54
pkmdemand	5.40E+12
EVRange	275
EVEfficiency_mult	1.25
BatteryEnergyDensity	0.25
SteelRecycledContent	0.085
LithiumRecycledContent	0.2155
CobaltRecycledContent	0.3515
AluminiumRecycledContent	0.246
CopperRecycledContent	0.265
NickelRecycledContent	0.2995
ManganeseRecycledContent	0.35
GraphiteRecycledContent	0.2985
LithiumBatteryComp	0.0179
AluminiumBatteryComp	0.1644
CopperBatteryComp	0.1598
NickelBatteryComp	0.144
ManganeseBatteryComp	0.152
GraphiteBatteryComp	0.2005
mEFreduce	0.5

The reference scenario is not intended as a forecast. It represents a consistent set of central

assumptions selected from the midpoint or best-estimate values of the uncertainty ranges and is used as a benchmark for one-at-a-time sensitivity analysis.

S4 Survival curves

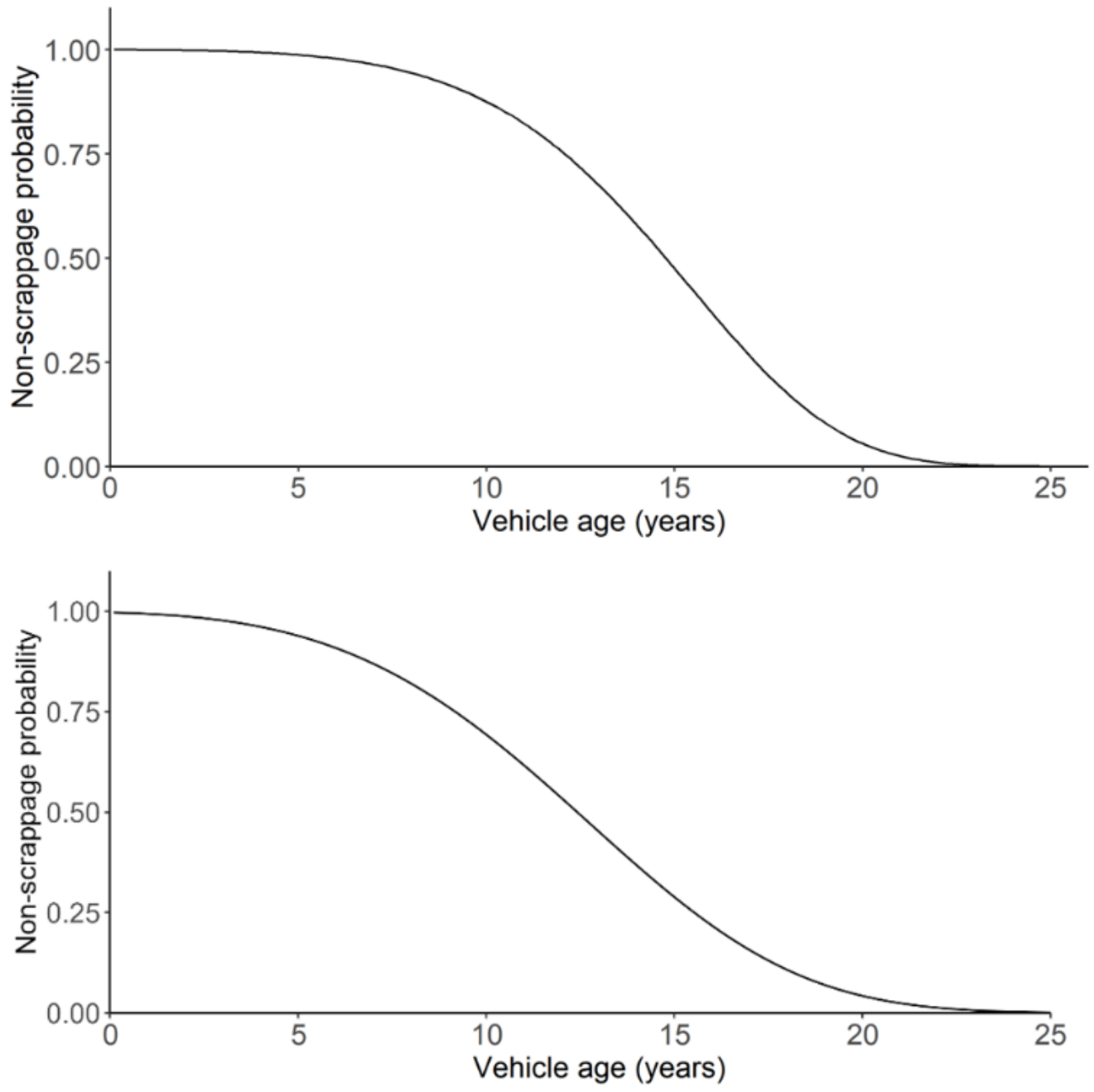

Figure S1 Survival curves of the UK[12] (top) and China (bottom). UK: b = 6.5, T = 22.5. China: b = 4, T = 18.

S5 Stock comparison

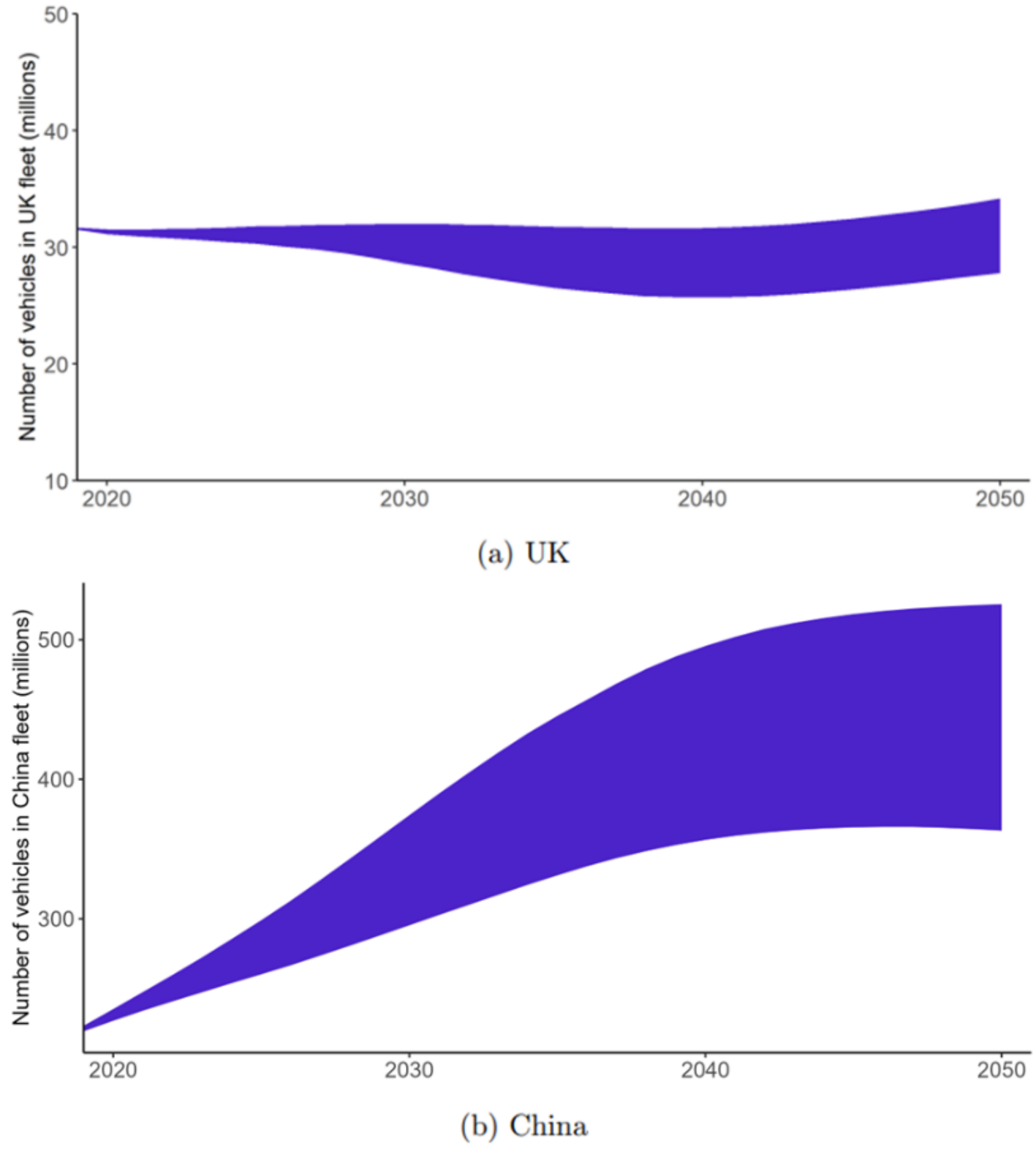

Figure S2 Vehicle stock comparison of China and UK[12]

S6 Material demands projection

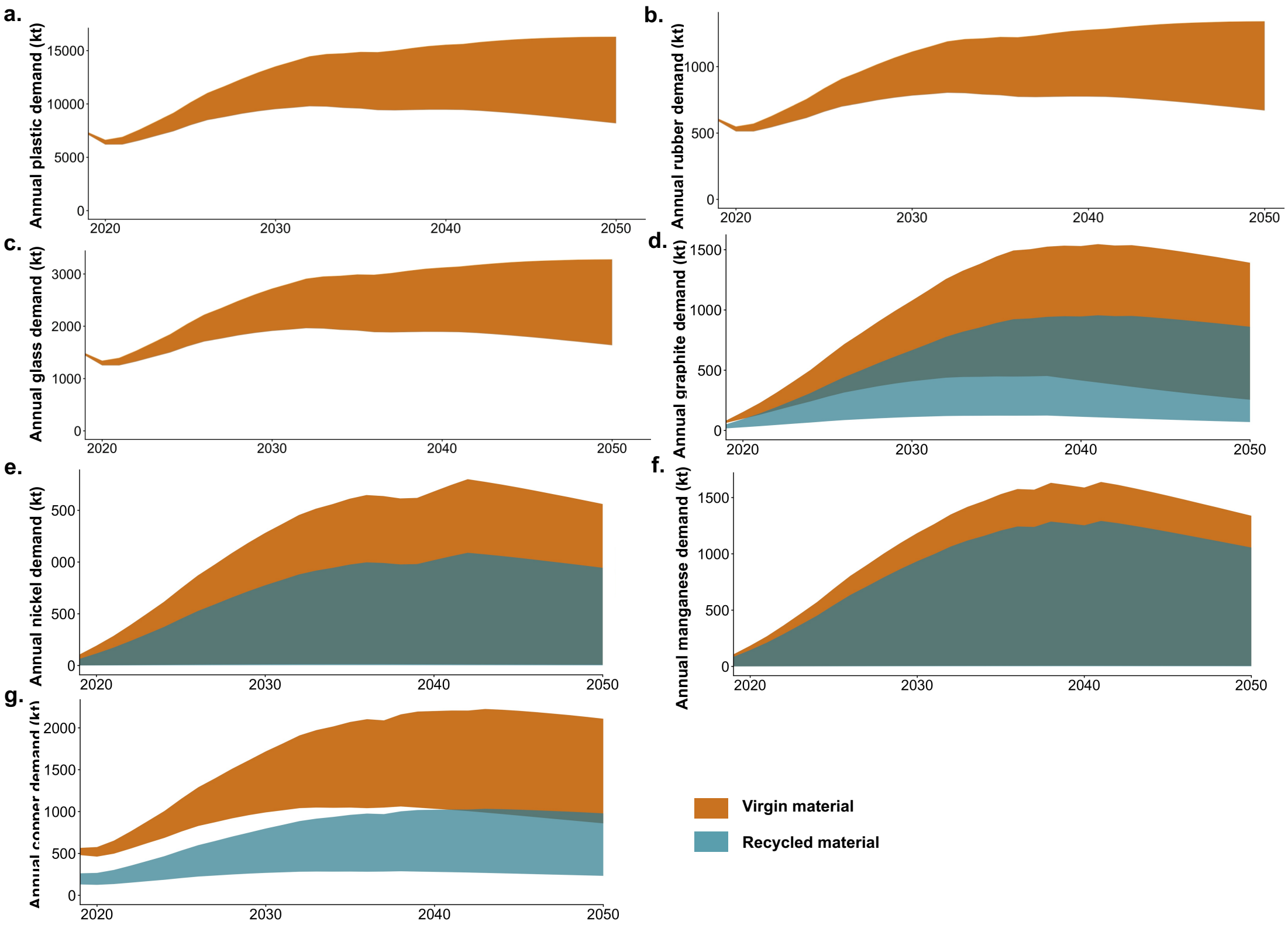

Figure S3 Annual material demands projection.

S7 WTW and embodied CO_2 emission projection

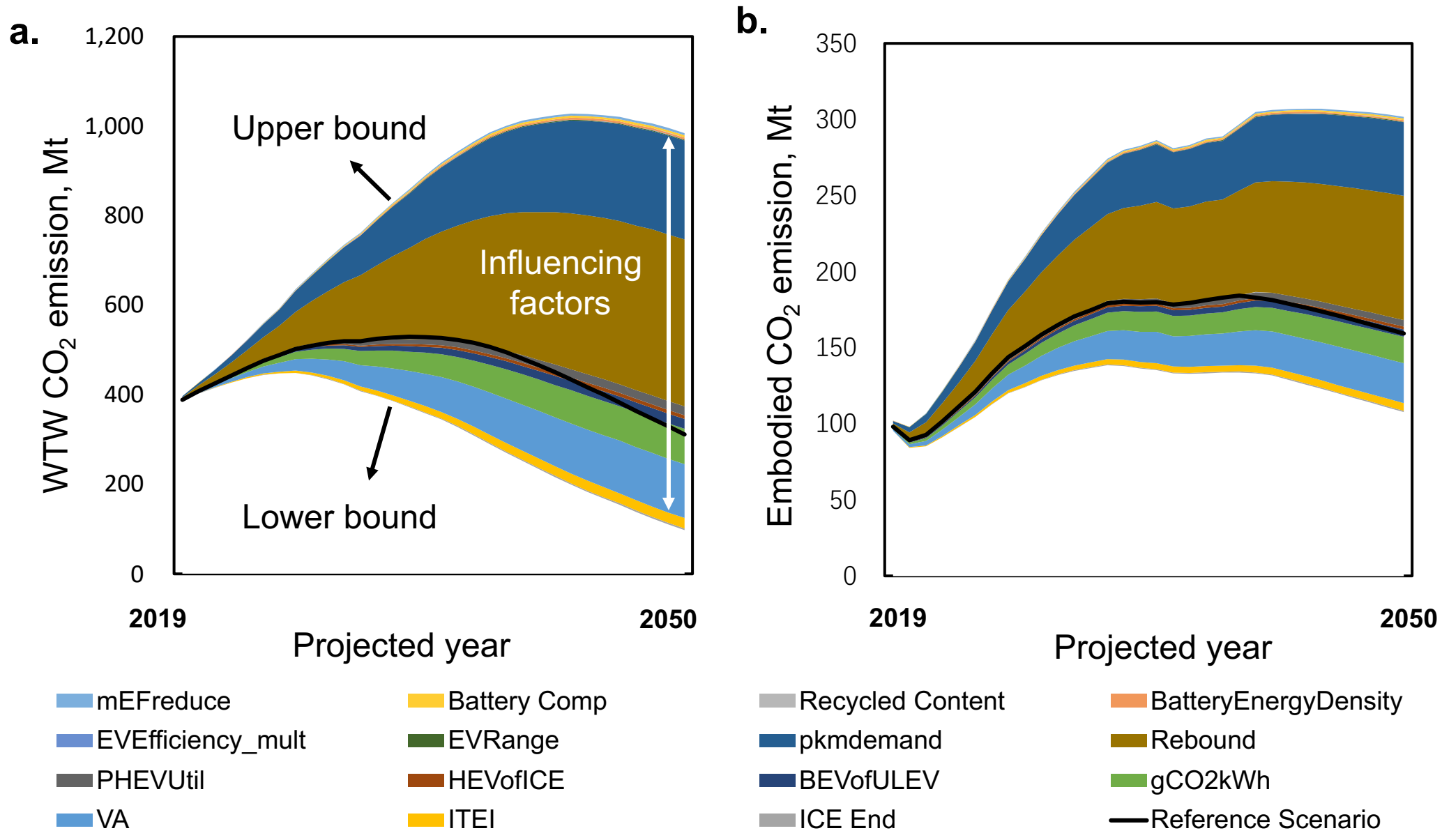

Figure S4 WTW and embodied CO_2 emission projection in China

S8 Sensitivity analysis

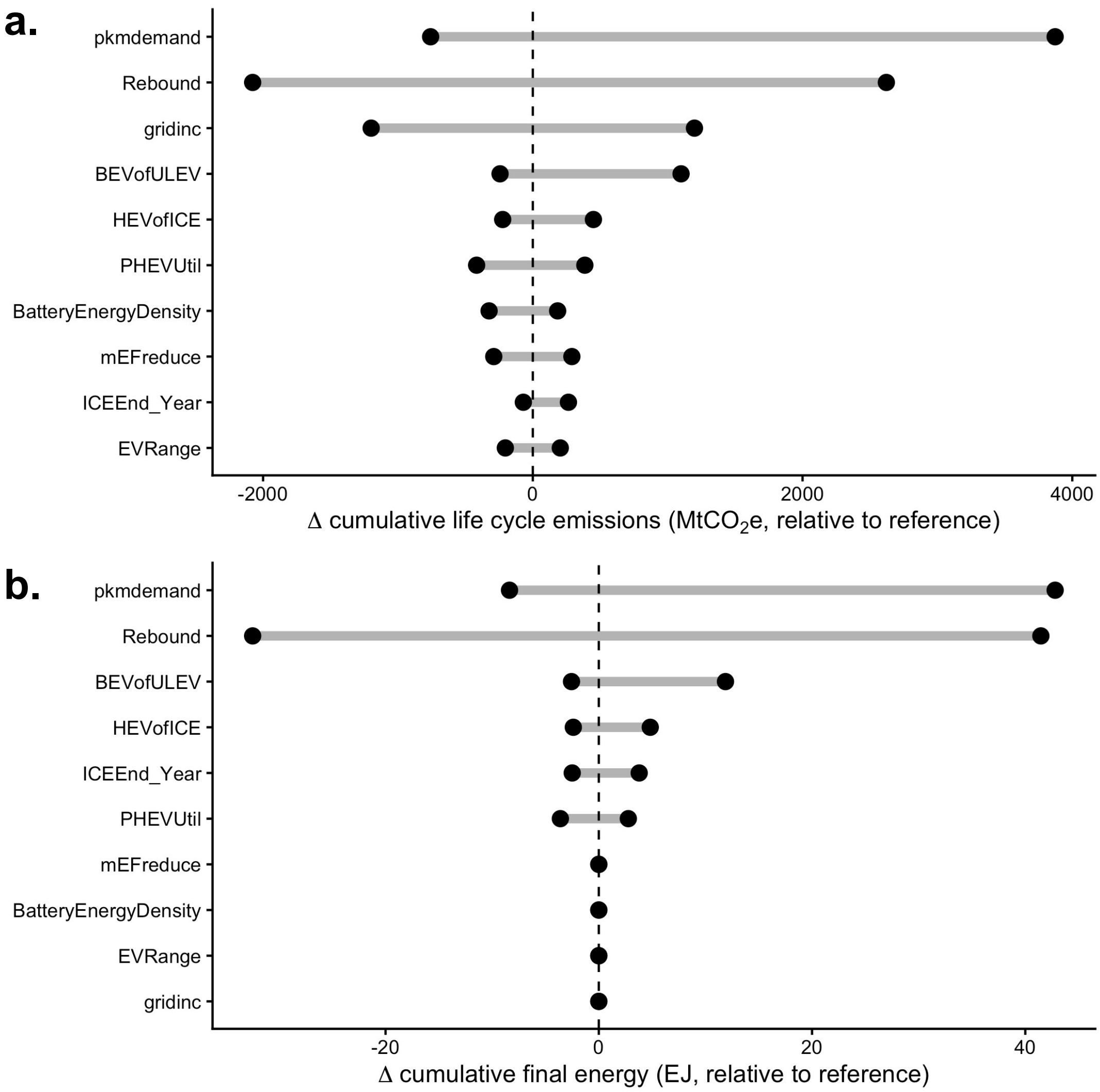

Figure S5 One-at-a-time sensitivity analysis around the reference scenario for cumulative life-cycle greenhouse gas emissions (top) and cumulative final energy demand (bottom) over 2019–2050. Each parameter is varied individually from its lower to upper bound while all others are fixed at reference values. Horizontal ranges show the resulting deviation from the reference scenario; black points mark low- and high-bound results, and the dashed vertical line indicates the reference scenario. Travel demand and rebound effect are the dominant drivers, with secondary contributions from electrification pathway variables. Unlike Sobol analysis, this one-at-a-time assessment is intended to illustrate the directional influence of individual parameters around the reference scenario and should not be interpreted as a measure of global parameter importance.

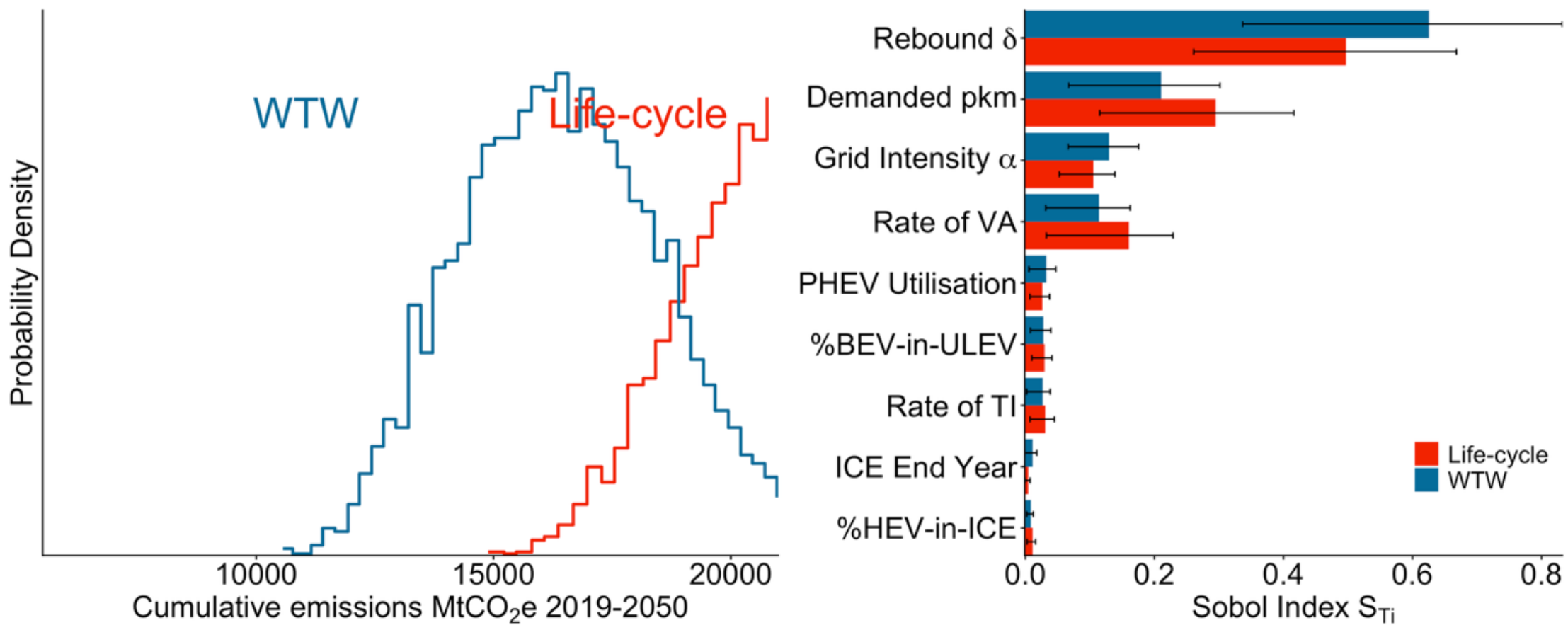

Figure S6 Distribution and sensitivity of cumulative greenhouse gas emissions for the China passenger vehicle fleet over 2019–2050. (Left) Probability density distributions of cumulative well-to-wheel (WTW) and full life-cycle CO_2 emissions derived from Monte Carlo simulations. (Right) Total-effect Sobol sensitivity indices (S_T) for cumulative life cycle CO_2 emissions, with error bars indicating standard errors. Higher Sobol indices indicate greater contribution of each input parameter to the variance in model outputs. Rebound effect and passenger travel demand dominate uncertainty, followed by grid carbon intensity and vehicle activity, while parameters related to powertrain shares and technology transitions have comparatively smaller effects.

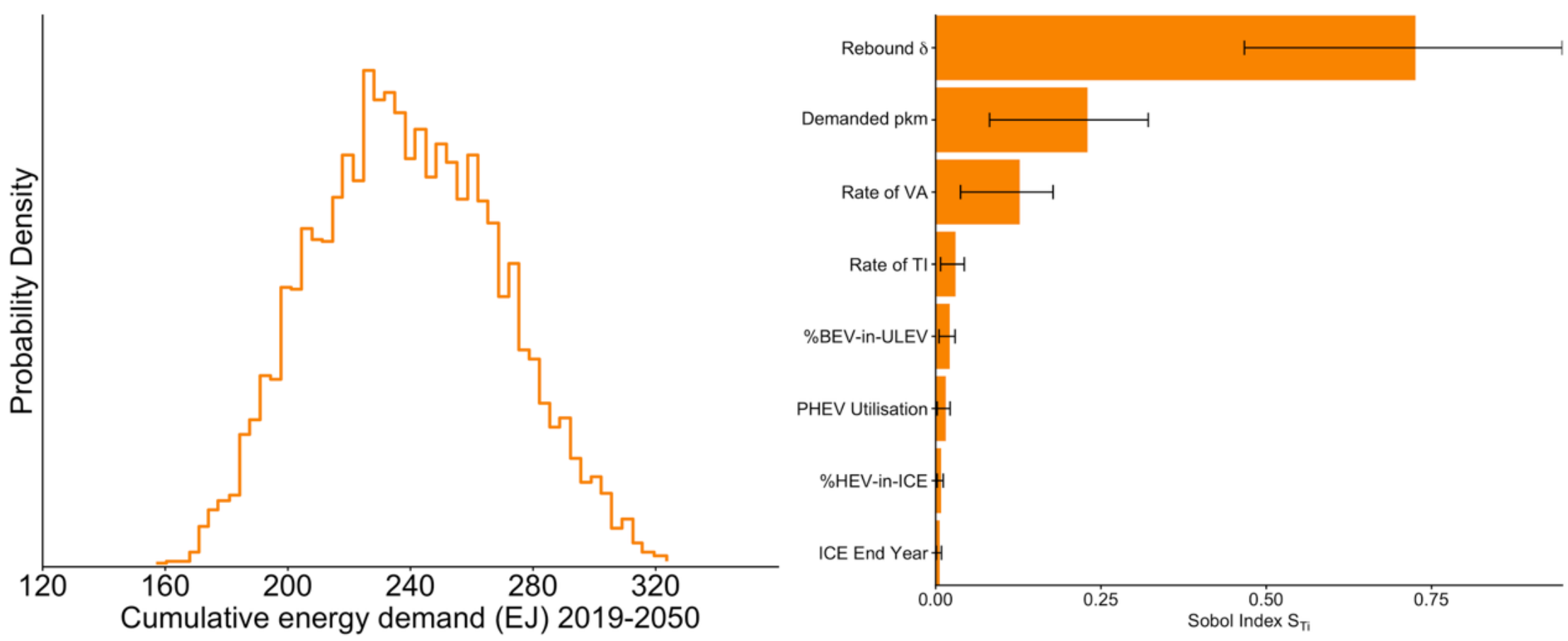

Figure S7 Distribution and sensitivity of cumulative energy use for China's passenger vehicle fleet over 2019–2050. (Left) Probability density distribution of cumulative energy use derived from Monte Carlo simulations. (Right) Total-effect Sobol sensitivity indices (S_T) for cumulative energy use, with error bars indicating standard errors. Larger Sobol indices indicate greater contributions of individual parameters to output variance. Rebound effect and passenger travel demand dominate uncertainty, followed by vehicle activity, whereas parameters related to powertrain shares and technology transitions contribute comparatively less.

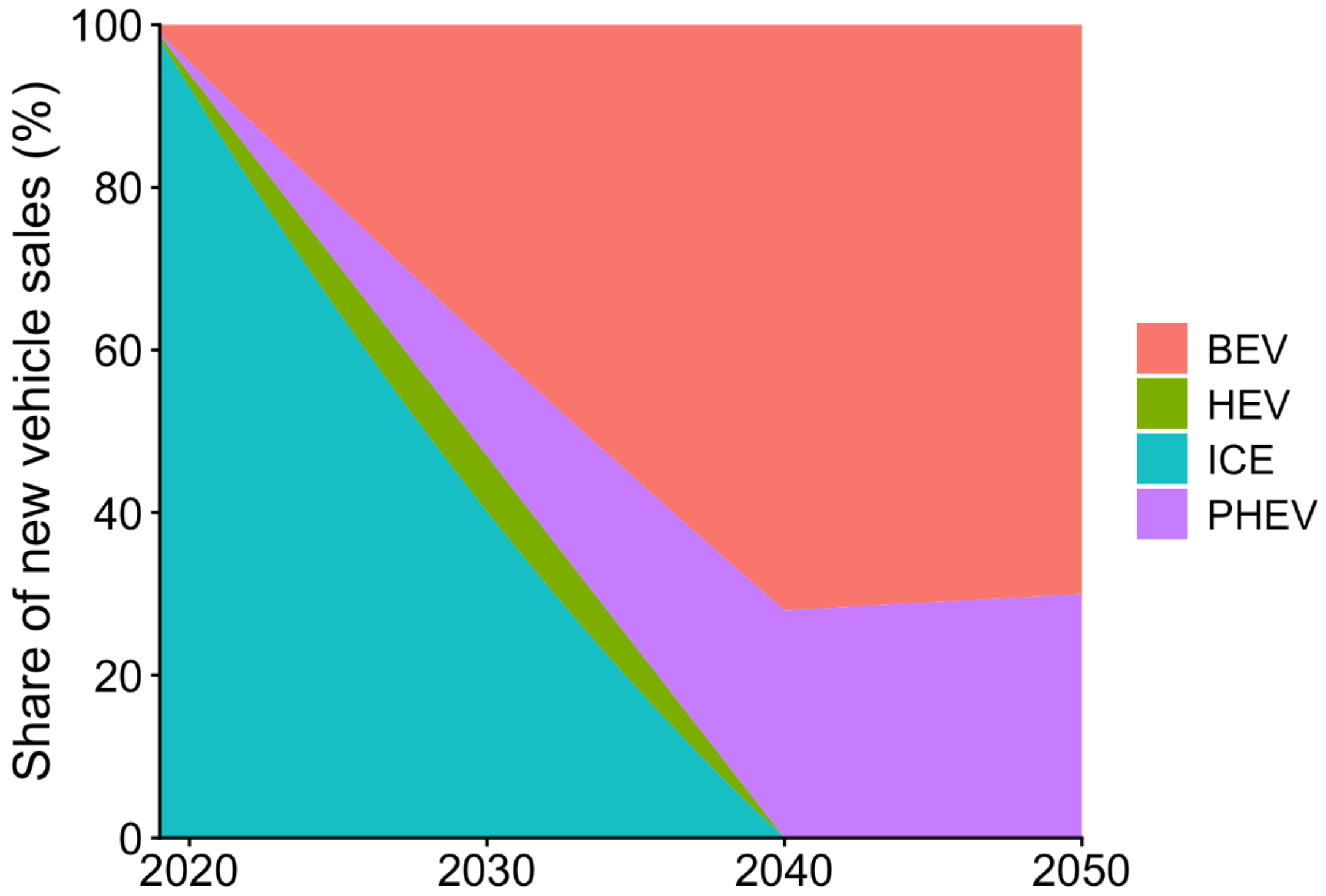

Figure S8 Assumed powertrain shares of new vehicle sales under the reference scenario (2019–2050). The figure shows the exogenously specified powertrain adoption pathway used in the reference scenario. ICE sales decline progressively and reach zero by the assumed ICE phase-out year (2038), after which new vehicle sales consist of BEVs and PHEVs only. Under the reference scenario, BEVs account for approximately 70% of new vehicle sales and PHEVs for approximately 30% by 2050.

S9 Algorithmic workflow of the China fleet projection model

Algorithm S1. Computational workflow of the integrated China passenger vehicle fleet projection model.

```
Algorithm S1. China fleet projection model

1. Read all deterministic model inputs
     • Vehicle stock and fleet composition
     • Survival curves
     • Vehicle mileage
     • Material inventories
     • Emission factors
     • Reference scenario assumptions

2. Define uncertain input variables
     • Sample all uncertain parameters from their specified
       uniform probability distributions (Table S2)
```

```
3. Monte Carlo uncertainty analysis

    For i = 1 to N simulations

        Sample all uncertain parameters

        Calculate:
            • Travel demand
            • Fleet stock evolution
            • Powertrain shares
            • Vehicle attributes
            • Battery weight
            • Material demand
            • Final energy demand
            • Well-to-wheel emissions
            • Embodied emissions
            • Life-cycle greenhouse gas emissions

        Store annual outputs (2019–2050)

    End For

4. Variance-based Sobol sensitivity analysis

    Generate Sobol A and B sampling matrices

    Construct hybrid sampling matrices

    Evaluate model for all Sobol samples

    Calculate first-order and total-effect Sobol indices

5. Reference scenario analysis

    Run the model using central parameter values

    Perform one-at-a-time parameter perturbations
```

```
6. Post-processing

    Calculate cumulative results

    Generate uncertainty ranges

    Produce figures and summary statistics
```

Reference

(1) Huo, H.; Wang, M. Modelling Future Vehicle Sales and Stock in China. *Energy Policy* **2012**, *43*. https://doi.org/10.1016/j.enpol.2011.09.063.

(2) China Automotive Technology and Research Center. *China Automobile Low Carbon Action Plan Research Report 2021*; 2021.

(3) Ou, S.; Yu, R.; Lin, Z.; Ren, H.; He, X.; Przesmitzki, S.; Bouchard, J. Intensity and Daily Pattern of Passenger Vehicle Use by Region and Class in China: Estimation and Implications for Energy Use and Electrification. *Mitig Adapt Strateg Glob Chang* **2020**, *25* (3). https://doi.org/10.1007/s11027-019-09887-0.

(4) Linn, J.; Shen, C. *The Effect of Income on Vehicle Demand: Evidence from China's New Vehicle Market*; Washington, DC, 2021.

(5) Zheng, Y.; Xu, H.; Jia, R. Endogenous Energy Efficiency and Rebound Effect in the Transportation Sector: Evidence from China. *J Clean Prod* **2022**, *335*. https://doi.org/10.1016/j.jclepro.2021.130310.

(6) Zhang, Y. J.; Peng, H. R.; Liu, Z.; Tan, W. Direct Energy Rebound Effect for Road Passenger Transport in China: A Dynamic Panel Quantile Regression Approach. *Energy Policy* **2015**, *87*. https://doi.org/10.1016/j.enpol.2015.09.022.

(7) IEA. *Data and statistics*. https://www.iea.org/data-and-statistics (accessed 2025-09-17).

(8) IEA. *Global fuel economy initiative 2021 report extract explore data*. https://www.iea.org/data-and-statistics/data-tools/global-fuel-economy-initiative-2021-data-explorer (accessed 2025-09-17).

(9) A. H. Jon Edmondson, Materials for electric vehicles: Electric motors, battery cells & packs, HV cabling 2020-2030," 2020. [Online]. Available: https://www.idtechex.com/en/research-report/materials-for-electric-vehicles-electric-motors-battery-cells-and-packs-hv-cabling-2020-2030/770.

(10) Liu, Min, Xin Sun, Ruixi Shen, et al. 'Real-World Life-Cycle Assessment of Industrial-Scale Lithium-Ion-Battery Hydrometallurgical Recycling'. Environmental Science & Technology 60, no. 8 (2026): 6203–14. https://doi.org/10.1021/acs.est.5c10922.

(11) Wernet, G., Bauer, C., Steubing, B., Reinhard, J., Moreno-Ruiz, E., and Weidema, B., 2016. The ecoinvent database version 3 (part I): overview and methodology. The International Journal of Life Cycle Assessment, [online] 21(9), pp.1218–1230. Available at: <http://link.springer.com/10.1007/s11367-016-1087-8>

(12) Poland, A. Modelling the Material Requirements and Emissions of Future UK Vehicles,

University of Cambridge, 2021.
